# Neddylation is required for perinatal cardiac development through stimulation of metabolic maturation

**DOI:** 10.1016/j.celrep.2023.112018

**Published:** 2023-01-19

**Authors:** Jianqiu Zou, Wenjuan Wang, Yi Lu, Juan Ayala, Kunzhe Dong, Hongyi Zhou, Jinxi Wang, Weiqin Chen, Neal L. Weintraub, Jiliang Zhou, Jie Li, Huabo Su

**Affiliations:** 1 Vascular Biology Center, Medical College of Georgia, Augusta University, Augusta, GA 30912, USA; 2 Key Laboratory of Protein Modification and Degradation, State Key Laboratory of Respiratory Disease, School of Basic Medical Sciences, Guangzhou Medical University, Guangzhou, Guangdong 511436, China; 3 Department of Neurology, The First Affiliated Hospital of Nanchang University, Nanchang, Jiangxi 330006, China; 4 Department of Pharmacology and Toxicology, Medical College of Georgia, Augusta University, Augusta, GA 30912, USA; 5 Department of Physiology, Medical College of Georgia, Augusta University, Augusta, GA 30912, USA; 6 Department of Medicine, University of Iowa, 200 Hawkins Drive, CBRB 2270B, Iowa City, IA 52242, USA; 7 Division of Cardiology, Department of Medicine, Medical College of Georgia, Augusta University, Augusta, GA 30912, USA; 8 Lead contact

## Abstract

Cardiac maturation is crucial for postnatal cardiac development and is increasingly known to be regulated by a series of transcription factors. However, post-translational mechanisms regulating this process remain unclear. Here we report the indispensable role of neddylation in cardiac maturation. Mosaic deletion of NAE1, an essential enzyme for neddylation, in neonatal hearts results in the rapid development of cardiomyopathy and heart failure. NAE1 deficiency disrupts transverse tubule formation, inhibits physiological hypertrophy, and represses fetal-to-adult isoform switching, thus culminating in cardiomyocyte immaturation. Mechanistically, we find that neddylation is needed for the perinatal metabolic transition from glycolytic to oxidative metabolism in cardiomyocytes. Further, we show that HIF1α is a putative neddylation target and that inhibition of neddylation accumulates HIF1α and impairs fatty acid utilization and bioenergetics in cardiomyocytes. Together, our data show neddylation is required for cardiomyocyte maturation through promoting oxidative metabolism in the developing heart.

## In brief

Zou et al. investigate the role of neddylation, a protein modification process, in the development and maturation of the heart during the perinatal stage. The results suggest that neddylation plays a crucial role in heart cell maturation and postnatal cardiac development by regulating the switch between glycolysis and oxidative metabolism.

## Graphical abstract

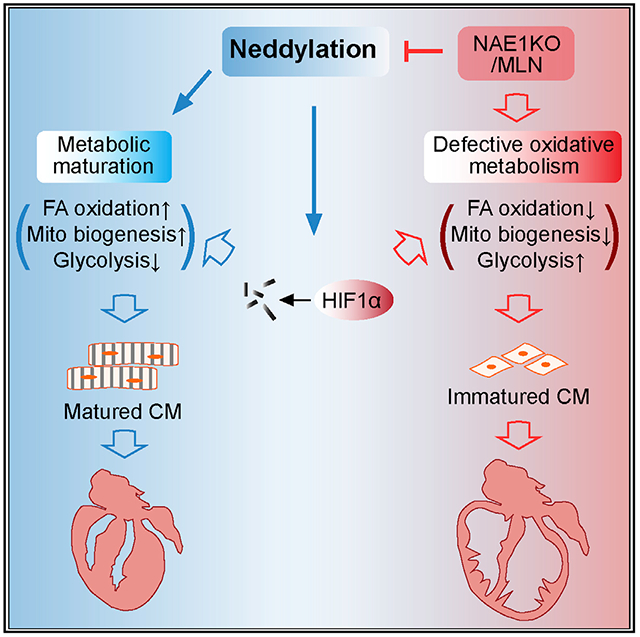

## INTRODUCTION

During development, the heart undergoes a series of profound structural, morphological, and functional changes until it ultimately develops into a functionally competent adult organ. From midgestation to adulthood, terminally differentiated cardiomyocytes (CMs) undergo progressive maturation processes, including sarcomeric protein isoform switching, transverse-tubule network development, electrophysiological maturation, cell-cycle withdrawal, hypertrophic growth, and metabolic reprogramming.^1,2^ Disruption of these critical maturation processes can lead to congenital heart disease or predisposition to cardiomyopathies and heart failure in adult life,^3–6^ emphasizing the significance of CM maturation in cardiac physiology and disease. Importantly, knowledge gained from cardiac maturation research might help to optimize approaches to promote the maturation of induced pluripotent stem cell (iPSC)-derived CMs used in cardiac regenerative medicine.^7^ Therefore, there is broad interest in understanding the regulatory mechanisms that underpin CM maturation.

Cardiac maturation involves crucial metabolic reprogramming during the transition from the fetal to the postnatal stage. Fetal hearts rely predominantly on glycolysis to generate ATP due to the hypoxic environment. In response to increased oxygen tension and altered nutrition sources postpartum, perinatal hearts primarily utilize fatty acid oxidation to meet their rapidly increased energy demands, which persists throughout life.^8^ This metabolic maturation is accompanied by the expansion of mitochondrial number and size,^9^ densification of mitochondrial cristae,^10^ upregulation of genes involved in fatty acid metabolism and oxidative phosphorylation,^11^ and downregulation of glycolytic genes.^12^ Perturbation of the developmental metabolic shift has been shown to cause heart failure and perinatal lethality in mouse models^13–17^ and in humans,^18^ highlighting a critical need to understand the upstream mechanisms governing this process. Over the past decade, considerable progress has been made in uncovering transcriptional regulators crucial for CM metabolic maturation, including hypoxia-inducible factor 1α (HIF1α),^6^ estrogen-related receptors α and γ (ERRα and ERRγ),^19^ and the peroxisome proliferator-activated receptor γ coactivator 1α/β/peroxisome proliferator-activated receptor (PGC1α/β/PPAR) axis.^5,14^ In particular, downregulation of HIF1α-mediated hypoxic signaling appears to be an important physiological switch driving metabolic reprogramming during cardiac maturation.^6,20^ While HIF1α is highly expressed in fetal hearts and is essential for embryonic heart development by sustaining CM proliferation,^21^ its level rapidly decreases from day 14.5 in murine embryonic hearts^6^ and after birth,^17,22^ and persistent activation of HIF1α in perinatal hearts impairs metabolic reprogramming, leading to heart failure and perinatal lethality.^6,20^

In addition to transcriptional mechanisms, posttranslational modifications (PTMs) provide another layer of regulation for cells to regulate diverse cellular processes. In eukaryotic cells, proteins are subjected to over 300 PTMs in response to external physical or chemical stimuli, which significantly expand the diversity of the proteome. PTMs result in rapid changes in protein conformation, subcellular localization, assembly/disassembly of multiple protein complexes, and binding affinity to DNA and proteins. By doing so, PTMs can subtly or dramatically alter protein activity and function without triggering *de novo* protein synthesis at the transcriptional level, thereby saving energy and material resources. Despite the increasing appreciation of PTMs, such as phosphorylation, methylation, acetylation, O-GlcNAcylation, ubiquitination, and SUMOylation, in cardiac development and pathophysiology,^23–26^ many more known PTMs remain to be investigated in heart tissues.

Neural precursor cell expressed, developmentally downregulated 8 (NEDD8) is a ubiquitin-like protein that covalently modifies target proteins in a way analogous to ubiquitination.^27^ Conjugation of NEDD8 to target proteins, termed neddylation, is catalyzed by a NEDD8-specific E1-E2-E3 enzyme system and can be reversed by NEDD8 proteases.^28–30^ By modulating the function of its various substrates, such as cullin proteins, neddylation regulates diverse cellular processes and multiple pathophysiological states, such as tumorigenesis,^31^ metabolic disorders,^32^ liver dysfunction,^33^ and neural development.^34,35^ We previously demonstrated that an intact NEDD8 pathway is essential for cardiac homeostasis in adulthood,^36–39^ and dysregulation of neddylation is associated with cardiomyopathies in human and murine hearts.^40^ More recently, we reported that neddylation is downregulated in the developing mouse heart after postnatal day 7, a time window when CMs start exiting the cell cycle.^41^ Deletion of NEDD8-activating enzyme 1 (NAE1), a regulatory subunit of the only NEDD8 E1 that is essential for NEDD8 activation and conjugation, in the developing heart via αMHC^Cre^ causes ventricular non-compaction and heart failure, which is at least in part attributable to inactivation of Yes-associated protein (YAP) signaling and CM proliferation arrest.^41^ While these findings demonstrate a critical role for neddylation in cardiac chamber development in embryonic hearts, the downregulation of neddylation in postnatal hearts drove us to determine whether neddylation is dispensable for postnatal cardiac development and whether neddylation has a role beyond regulating CM proliferation.

In this report, we describe a series of studies designed to determine the role of neddylation in postnatal hearts. Using strategies to conditionally delete the gene encoding NAE1 in mice, we demonstrate that neddylation is necessary for proper control of CM metabolic transition and for normal peri- and postnatal cardiac maturation. Furthermore, we identify that neddylation is required to suppress HIF1α signaling by directly modifying HIF1α and its ubiquitin ligase cullin 2 (Cul2).

## RESULTS

### Postnatal deletion of NAE1 induces dilated cardiomyopathy and heart failure

**Figure 1.**
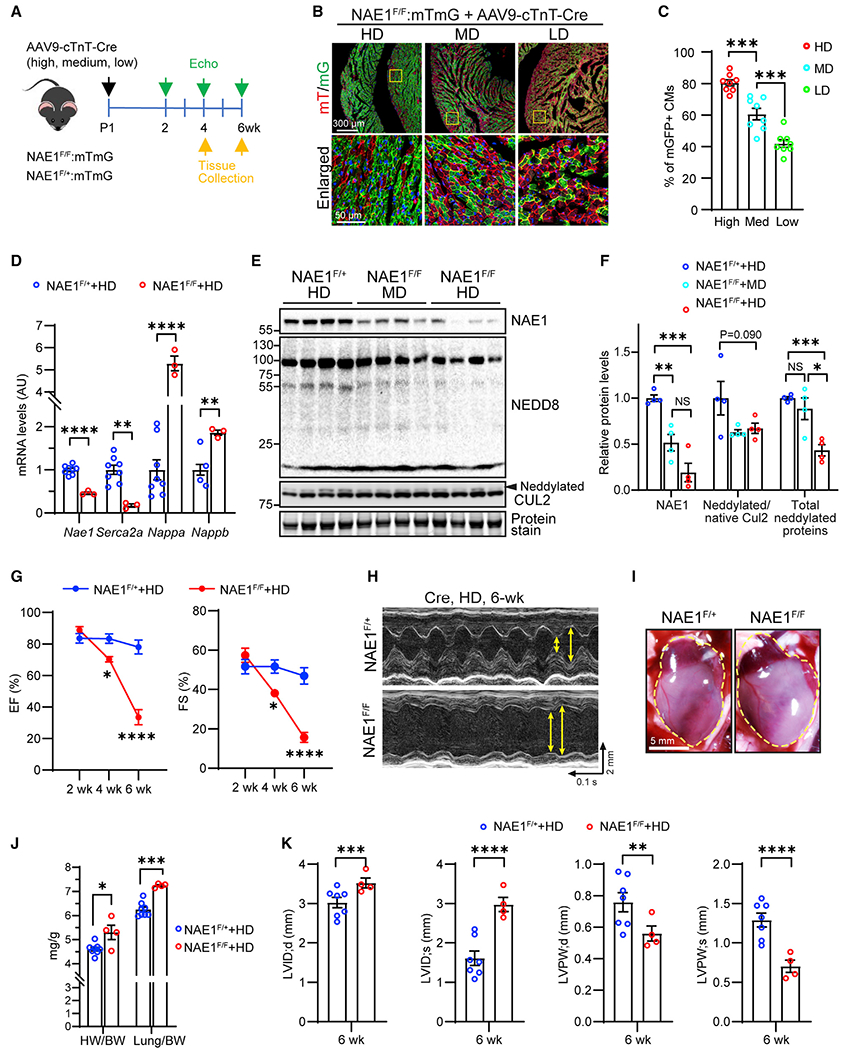
Deletion of NAE1 in postnatal heart causes heart failure (A) Scheme depicting experimental timeline. Neonatal NAE1^Flox/+^ and NAE1^Flox/Flox^ pups carrying the mTmG reporter were injected with AAV9-cTnT-Cre at high (HD), medium (MD), and low (LD) doses subcutaneously and subjected to echocardiography (Echo) and tissue collection at indicated times. (B) Representative confocal images of mGFP^+^ (mG) and mtdTomato^+^ (mT) CMs in the heart 4 weeks post AAV injection. Part of the ventricular myocardium (top, scale bar, 300 μm) and enlarged box areas (bottom, scale bar, 50 μm) are shown. (C) Quantification of the percentage of mGFP^+^ CMs in AAV-infected hearts; n = 8 fields from three hearts/dose. (D) Quantitative real-time PCR analysis of indicated genes in hearts of 4-week-old mice receiving HD AAVs; n = 8 vs. 3. (E and F) Western blot analysis (E) and quantification (F) of indicated proteins in hearts of 4-week-old mice infected with HD or MD AAVs; n = 4 vs. 4 vs. 4. (G) Echocardiography showing temporal changes in ejection fraction (EF) and fractional shortening (FS) in mice receiving HD AAVs; n = 7 vs. 4. (H) Echocardiography B-mode images in 6-week-old mice post HD AAV injection. Double-headed arrows mark the ventricular chamber diameter at systole and diastole, respectively. (I) Gross morphology of 6-week mouse hearts infected with HD AAVs. Scale bar, 5 mm. (J) Heart weight (HW)-to-body weight (BW) ratio and lung weight-to-BW ratio; n = 8 vs. 4. (K) Ventricular chamber size and wall thickness at 6 weeks post HD AAV injection; n = 7 vs. 4. LVID, left-ventricle (LV) internal diameter; LVPW, LV posterior wall thickness; d, diastolic state; s, systolic state. *p < 0.05; **p < 0.01; ***p < 0.001; ****p < 0.0001; NS, not significant. Error bars indicate SEM. For (F), two-way ANOVA followed by multiple comparison was performed. All other statistical analysis was performed by one-way ANOVA followed by multiple comparison.

Perinatal lethality of αMHC^Cre^-driven NAE1 knockout^41^ prevents using this mouse line to investigate the role of neddylation in postnatal cardiac development. We employed adeno-associated virus 9 (AAV9) expressing Cre recombinase under the control of the cardiac troponin T (cTnT) promoter^42^ to generate mosaic cardiac-specific NAE1 knockout mice. The AAV-Cre was injected into neonatal NAE1^F/+^ and NAE1^F/F^ mice carrying a lineage-tracing reporter Rosa26^mTmG^ allele^43^ (Figure 1A), which labels Cre-expressing cells with membrane-bound GFP (mG) and non-expressing cells with membrane-bound Tomato (mT) (Figures 1B and S1A). High-dose AAV-Cre (5 × 10^11^ viral genome copies [GC]/pup) transduced about 80% of CMs and significantly reduced NAE1 transcripts and proteins, leading to a significant decrease in total neddylated proteins and neddylated Cul2, a well-known NEDD8 substrate, in NAE1^F/F^ hearts compared with NAE1^F/+^ littermates (Figures 1D–1F), confirming inhibition of neddylation. Cardiac phenotypes of these mice were characterized by temporal echocardiography and morphological and gravimetric analyses. Compared with littermate NAE1^F/+^ mice, high-dose AAV-Cre induced progressive dilated cardiomyopathy and eventually heart failure in NAE1^F/F^ mice by 6 weeks of age, evidenced by enlarged heart size, increased heart weight-to-body weight and lung weight-to-body weight ratios, increased left-ventricular chamber size, reduced left-ventricular wall thickness, and gradually deteriorating left-ventricular ejection fraction and fractional shortening (Figures 1G–1K). Quantitative real-time PCR demonstrated the upregulation of the cardiac stress markers *Nppa* and *Nppb* and the downregulation of *Serca2a* (Figure 1D). Histological analyses showed enlarged heart size and prominent cardiac fibrosis in NAE1^F/F^ hearts (Figures S1B and S1C). Terminal deoxynucleotidyl transferase dUTP nick-end labeling (TUNEL) staining, Evans blue dye (EBD) infiltration assay, and immunoblotting of cleaved caspase 3 did not identify evidence of pronounced CM cell death in 4- and 6-week-old NAE1^F/F^ hearts (Figures S1D–S1F). Taken together, these data demonstrate that neddylation is indispensable for normal postnatal cardiac development.

### Deletion of NAE1 impairs CM maturation

Since the deletion of NAE1 occurred in the critical time window of CM maturation, we hypothesized that disruption of CM maturation underlies the pathogenesis of cardiomyopathy in NAE1^F/F^ hearts receiving high-dose AAV-Cre. To rule out confounding effects secondary to pathological cardiac remodeling, we titrated AAV-Cre doses, which led to detection of Cre activity in ~60% and 40% of CMs at medium (0.5 × 10^11^ viral GC/pup) and low (0.25 × 10^11^ viral GC/pup) doses, respectively (Figure 1C). Neither dose caused discernible cardiac remodeling in NAE1^F/F^ mice, evidenced by comparable fibrotic areas, cardiac morphometric parameters, ejection fraction, and left-ventricular mass measured by echocardiography between NAE1^F/F^ and NAE1^F/+^ mice at 4 weeks of age (Figures S1G and S1H). Thus, these hearts were used to assess the impact of NAE1 deletion on CM maturation.

**Figure 2.**
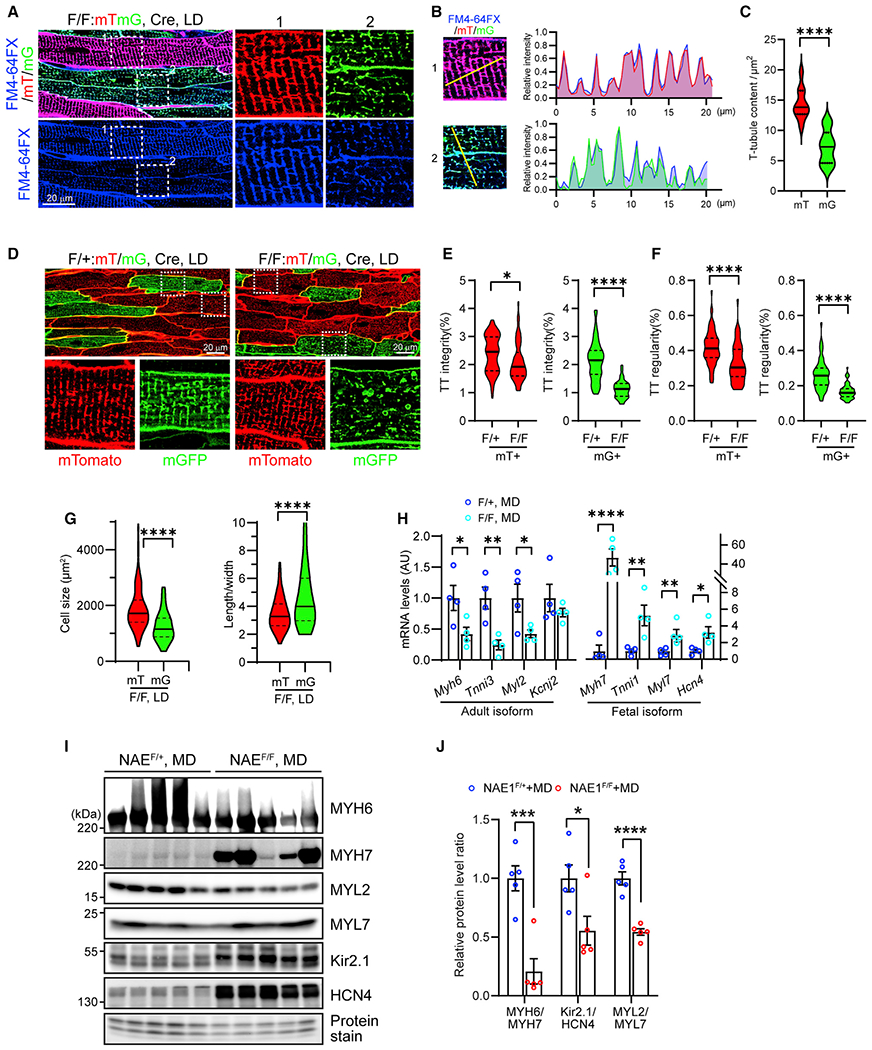
Inhibition of neddylation impairs CM maturation (A) *In situ* confocal imaging of surface myocardium in FM 4-64FX-stained 4-week-old NAE1^F/F^ hearts infected with LD AAVs. Boxed areas from mT^+^ (1) and mG^+^ (2) CMs are enlarged to show T-tubule patterns. (B) Histogram of fluorescence intensity of the yellow line-marked regions in boxed areas in (A), scale bar, 20 μm. Note the overlap of FM 4-64FX signals (blue) with mT (red) and mG (green) signals. (C) Quantification of T-tubule contents in mT^+^ and mG^+^ CMs in hearts of 4-week-old NAE1^F/F^ mice infected with LD AAVs. (D) *In situ* confocal imaging of surface myocardium from hearts of 4-week-old NAE1^F/+^ and NAE1^F/F^ mice infected with LD AAVs. Scale bar, 20 μm. Boxed areas are enlarged and shown at bottom. (E and F) Quantification of T-tubule integrity (E) and regularity (F). (G) Cell size and geometry of CMs isolated from 4-week-old mouse hearts infected with LD AAVs. (H) Quantitative real-time PCR analysis of indicated genes in hearts from 4-week-old mice infected with MD AAVs. (I) Western blot analysis of indicated proteins in hearts from 4-week-old mice infected with MD AAVs. (J) Ratios of the adult to fetal isoforms of the indicated proteins in (I). *p < 0.05; **p < 0.01; ***p < 0.001; ****p < 0.0001. Error bars indicate SEM. For violin plots in (C), (E), (F), and (G), n = 120 vs. 120 fields of view within n = 3 vs. 3 biological replicates, analyzed by unpaired Student’s t test. For (H), n = 4 vs. 4 biological replicates. For (J), n = 5 vs. 5 biological replicates. Statistical analysis was by one-way ANOVA followed by multiple comparison.

To visualize T-tubule organization in live CMs *in situ*, we labeled T tubules with the plasma membrane dye FM 4-64FX through retrograde heart perfusion and performed *in situ* confocal imaging.^44^ We observed the T tubules to be drastically disorganized in mG^+^ CMs in NAE1^F/F^ hearts infected with low-dose AAV-Cre, while those in neighboring mT^+^ CMs remained well aligned (Figure 2A). Quantification of FM 4-64FX fluorescence intensity showed a significant reduction in T-tubule contents in mG^+^ CMs vs. mT^+^ CMs (Figure 2C). Interestingly, we noted that the mG and mT signals perfectly matched those of FM 4-64FX (Figure 2B), likely due to the membrane-targeting sequence. Immunostaining of the T-tubule protein junctophilin-2 (JPH2) further confirmed that both mT and mG colocalize with JPH2 (Figures S2A and S2B), suggesting that the mT/mG reporter faithfully traces the T-tubule network. Examining mT/mG patterns in 4-week-old NAE1^F/+^ and NAE1^F/F^ hearts receiving low-dose AAV-Cre revealed a remarkable disruption of T tubules in mG^+^ CMs from NAE1^F/F^ hearts, but not in mT^+^ CMs from NAE1^F/F^ hearts or in mG^+^ or mT^+^ CMs from NAE1^F/+^ hearts (Figure 2D). Quantification of T-tubule patterns showed a significant reduction of T-tubule integrity and regularity in mG^+^ CMs from NAE1^F/F^ hearts (Figures 2E and 2F). Together these data suggest that neddylation is essential for T-tubule organization.

In addition to T-tubule development, CM maturation is accompanied by CM growth and switch of fetal to adult gene expression.^1^ We isolated CMs from low-dose AAV-treated NAE1^F/F^:mTmG hearts and found that mG^+^ CMs exhibited decreased cell area and increased length-to-width ratio compared with mT^+^ CMs, indicating an important role for neddylation in CM growth (Figure 2G). We further assessed the expression of the fetal and adult isoforms of genes involved in sarcomere and electrophysiological maturation. Quantitative real-time PCR analysis of medium-dose AAV-treated hearts revealed a significant downregulation of sarcomeric adult isoforms, including *Myh6, Tnni3,* and *Myl2,* and potassium channel gene *Kcnj2* in NAE1^F/F^ hearts compared with NAE1^F/+^ hearts. In contrast, the fetal isoforms of these genes, such as *Myh7, Tnni1, Myl7,* and *Hcn4,* were drastically upregulated (Figure 2H). Immunoblotting confirmed these changes at the protein level in medium-dose AAV-treated hearts (Figures 2I and 2J). Thus, these data identify an essential role for neddylation in CM maturation.

### Transcriptome analysis reveals defective maturation and metabolic dysregulation in NAE1-deficient hearts

**Figure 3.**
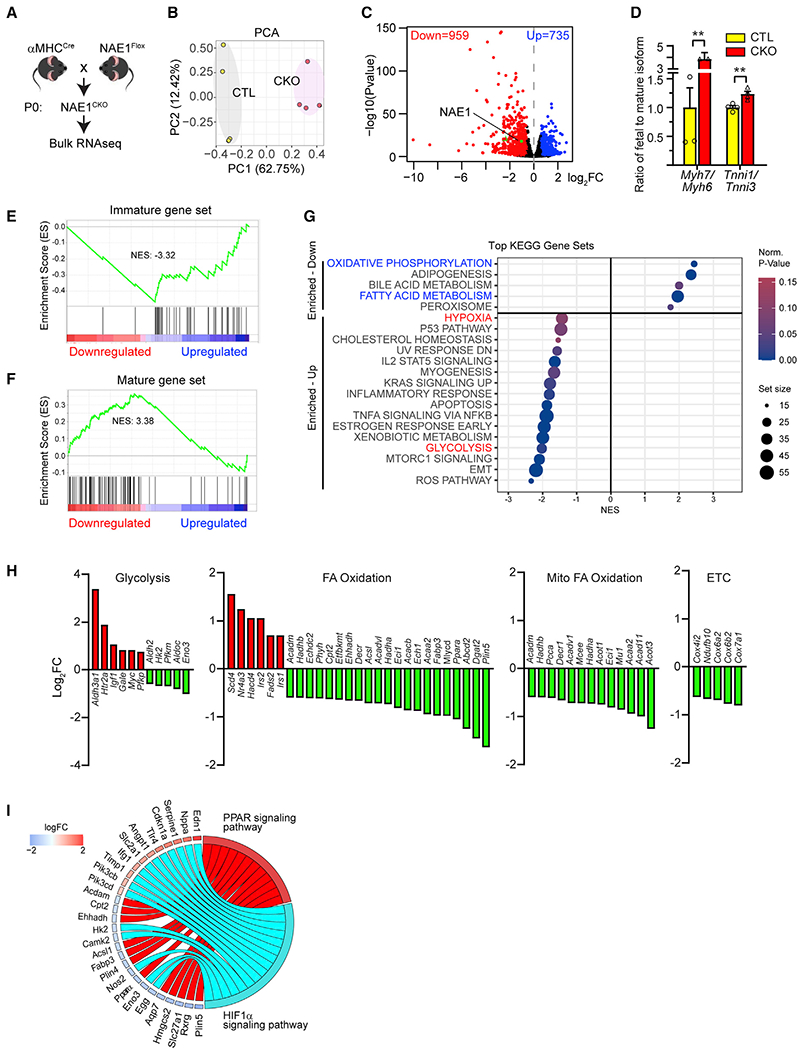
Transcriptome analysis reveals metabolic derangement in neonatal NAE1-deficient heart (A) Scheme showing experiment design. (B) Principal-component analysis (PCA) of RNA-seq replicates. (C) Volcano plot showing significantly downregulated (blue) and upregulated (red) genes in hearts from NAE1^CKO^ mice (fold change [FC] > 1.5 or <−1.5 and p_adj_ <0.05). (D) Ratios of fetal to adult isoforms in neonatal hearts from control and NAE1^CKO^ mice (from RNA-seq data); n = 4 vs. 4 biological replicates. (E and F) Gene set enrichment analysis of immature (E) and mature (F) gene sets. (G) Gene set enrichment analysis of differentially expressed genes in hearts from NAE1^CKO^ mice showed enrichment of the indicated KEGG pathways. (H) Bar graphs showing dysregulation of the indicated metabolic genes in hearts from NAE1^CKO^ mice. (I) Chord plots showing enrichment of dysregulated metabolic genes in the PPAR and HIF1α signaling pathways. **p < 0.01. Error bars indicate SEM. For (D), one-way ANOVA followed by multiple comparison was performed.

To gain insights into how neddylation regulates CM maturation, we sought to conduct transcriptional profiling. Analysis of mouse hearts with AAV-Cre-mediated mosaic NAE1 deletion may underestimate the transcriptional changes, and isolation and sorting of viable adult mG^+^ and mT^+^ CMs from NAE1^F/F^ hearts is challenging. Therefore, we chose to analyze the transcriptomes of αMHC^Cre^-driven neonatal NAE1 knockout hearts (NAE1^CKO^) (Figure 3A). Principal-component analysis revealed patterns distinguished between NAE1^CKO^ and littermate control groups (Figure 3B). Differential gene expression analysis identified 959 downregulated and 735 upregulated genes, with *Nae1* being one of the top downregulated genes (Figure 3C, Table S1). Consistent with the findings in AVV-infected hearts, the ratios of fetal to mature myosin heavy chain isoforms (*Myh7* to *Myh6*) and troponin I isoforms (*Tnni1* to *Tnni3*) were also significantly increased in NAE1^CKO^ hearts (Figure 3D). To further analyze the global impact of NAE1 deletion on the CM maturation status, gene set enrichment analysis (GSEA) was performed to assess the enrichment of differentially expressed genes among the previously identified mature (293 genes) and immature (354 genes) gene sets common in human and mouse.^45^ Genes upregulated in NAE1-deficient hearts were highly enriched for immature genes (normalized enrichment score [NES] = −2.32; Figure 3E), while downregulated genes were highly enriched for mature genes (NES = 3.38; Figure 3F). GSEA of the differentially expressed genes among the top 100 neonatal- or adult-specific genes identified in mouse heart^3^ also revealed a similar trend: upregulated genes were enriched for neonatal genes (NES = −1.04; Figures S3A and S3C), while downregulated genes were enriched for adult genes (NES = 1.31; Figures S3B and S3D). These data suggest that inhibition of neddylation results in persistent expression of genes associated with immaturity and inactivation of the transcriptional network required for CM maturation.

We next analyzed the RNA-sequencing (RNA-seq) data to identify biological processes enriched within the differentially expressed genes in NAE1-deficient hearts. Interestingly, the downregulated genes were highly enriched for oxidative phosphorylation and fatty acid metabolism, while the upregulated genes were enriched for more diverse cellular processes, including hypoxia signaling and glycolysis (Figure 3G). Specifically, many genes involved in fatty acid oxidation, mitochondrial fatty oxidative oxidation, and electron transportation chain were significantly downregulated in NAE1-deficient hearts (Figures 3H and S3E), whereas genes involved in glucose transport and glycolysis were either up- or downregulated (Figure S3F). Interestingly, many of these dysregulated metabolic genes were enriched in PPARα and HIF1α signaling (Figure 3I), suggesting their potential involvement in metabolic dysregulation. Screening of a list of key metabolic transcriptional regulators showed a significant downregulation of PERM1 and PPARα, but not others, such as HIF1α, in NAE1-deficient hearts (Figure S3G). These data identify a link between neddylation and cardiac metabolic maturation.

### Defective oxidative metabolism in NAE1-deficient hearts

**Figure 4.**
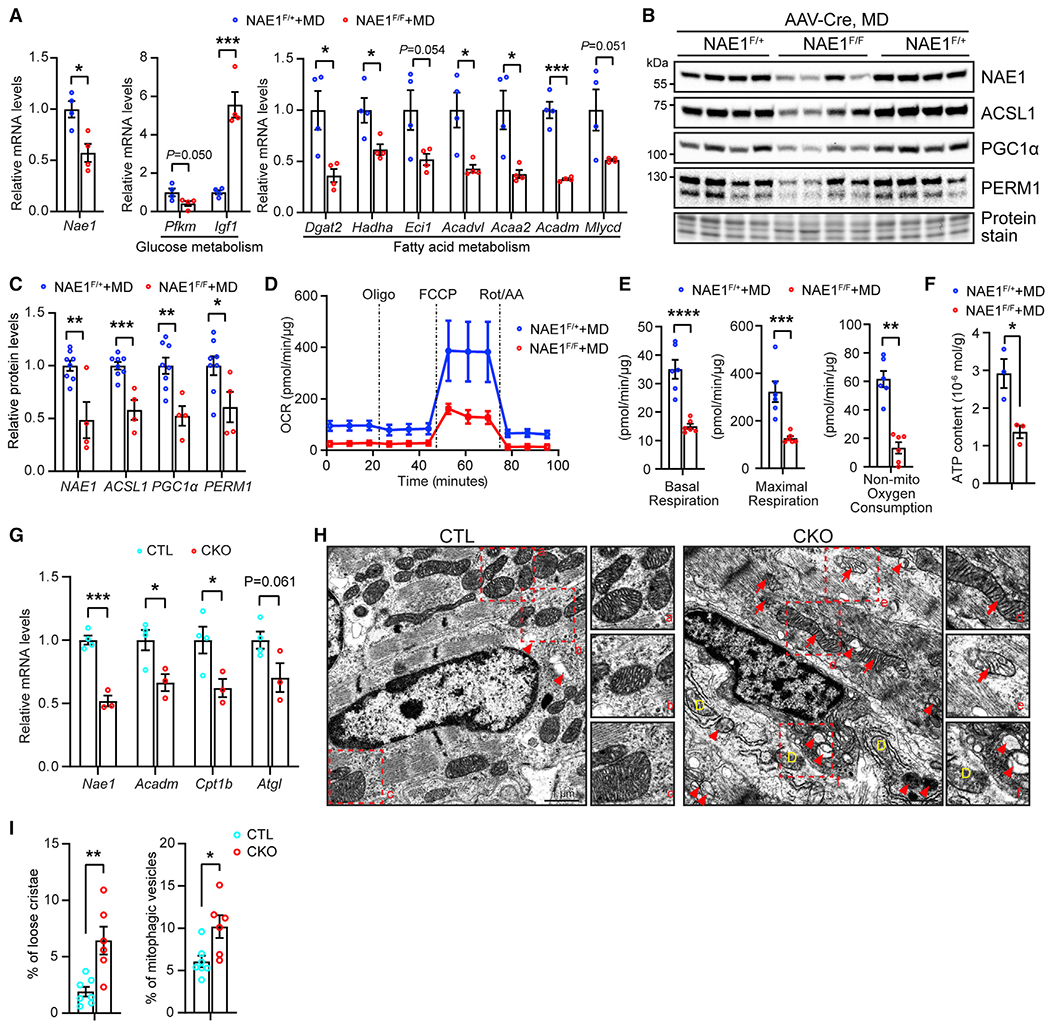
Fatty acid metabolic and mitochondrial dysfunction in hearts from NAE1-deficient mice (A–F) Hearts from 4-week-old mice receiving MD AAVs at P0 were used for the analyses. (A) Quantitative real-time PCR analysis of indicated metabolic genes; n = 4 vs. 4. (B and C) Western blots (B) and quantification (C; n = 8 vs. 4) of indicated proteins. (D and E) Seahorse analysis of CMs isolated from hearts of 4-week-old mice. Oxygen consumption rate (OCR) (D) and quantification of basal respiration, maximal respiration, and non-mitochondrial oxygen consumption are shown (E); n = 6 vs. 6. (F) ATP content of indicated heart tissues per gram protein; n = 3 vs. 3. (G and H) Hearts from neonatal CTL and NAE1^CKO^ mice at P1 were subjected to the analyses. (G) Quantitative real-time PCR analysis of the indicated genes; n = 4 vs. 3. (H) Transmission electron microscopy (TEM) images. Boxed areas are enlarged in (a)–(f) to show mitochondrial structures. Arrows mark mitochondria with loose cristae; arrowheads point to mitophagic vesicles; yellow D indicates degenerating mitochondria. Scale bar, 1 μm. (I) Quantification of the percentage of mitochondria with loose cristae or mitophagic vesicles; n = 7 vs. 6. *p < 0.05; **p < 0.01; ***p < 0.001; ****p < 0.0001. Error bars indicate SEM. All statistical analyses were performed by one-way ANOVA followed by multiple comparison.

Perinatal metabolic transition from glycolysis to oxidative metabolism is essential for CM maturation and postnatal cardiac development.^8,46^ Quantitative real-time PCR analysis showed that several genes involved in fatty acid utilization, such as *Dgat2, Hadha, Mlycd,* and *Eci1,* were significantly downregulated in medium-dose AAV-Cre-infected NAE1^F/F^ hearts compared with NAE1^F/+^ hearts, whereas *Igf1*, a gene that stimulates glucose utilization, was markedly upregulated (Figure 4A). In contrast, we did not observe a significant change in stress markers *(Nppa, Nppb,* and *Myh6)* (Figure S4A), consistent with the lack of pronounced cardiac dysfunction in these hearts (Figure S1C). Moreover, western blotting revealed a decrease in the enzyme regulating fatty acid utilization (acyl-CoA synthetase long chain family member 1, ACSL1) and proteins involved in mitochondrial biogenesis (PGC1α and PERM1)^47^ in medium-dose AAV-Cre-infected NAE1^F/F^ hearts (Figure 4B and 4C). Measurement of respiration function of isolated cardiac mitochondria showed a substantial reduction in basal and maximal mitochondrial respiration in NAE1^F/F^ hearts (Figures 4D and 4E). In addition, the ATP content in NAE1^F/F^ hearts also decreased to nearly half of the levels in NAE1^F/+^ hearts (Figure 4F). Since medium-dose AAV-Cre deletes NAE1 in ~60% of CMs, these metabolic changes may be underestimated, highlighting the impact of neddylation on cardiac metabolism.

Cardiac metabolism switching from glycolysis to oxidative metabolism starts at midgestation.^6^ We sought to determine whether NAE1^CKO^ affects cardiac metabolic function. Quantitative real-time PCR analysis showed that NAE1^CKO^ led to dramatically decreased mRNA levels of fatty acid oxidation genes, including *Acadm, Cpt1b,* and *Atgl* (Figure 4G). Ultra-structural analysis revealed a significant increase in lipid droplets in mutant CMs (Figures S4B and S4C). Oil red O (ORO) staining revealed increased accumulation of lipid droplets in NAE^CKO^ hearts compared with littermate control hearts (Figure S4D). Meanwhile, NAE1-deficient CMs also displayed a remarkable increase in degenerating/immature mitochondria with loose and fragmented cristae and mitophagic vesicles (Figures 4H and 4I), suggesting mitochondrial dysfunction. Thus, the *in vivo* findings from NAE1-deficient hearts support a critical role for neddylation in instigating cardiac oxidative metabolism.

### Inhibition of neddylation impairs fatty acid utilization in cultured CMs

**Figure 5.**
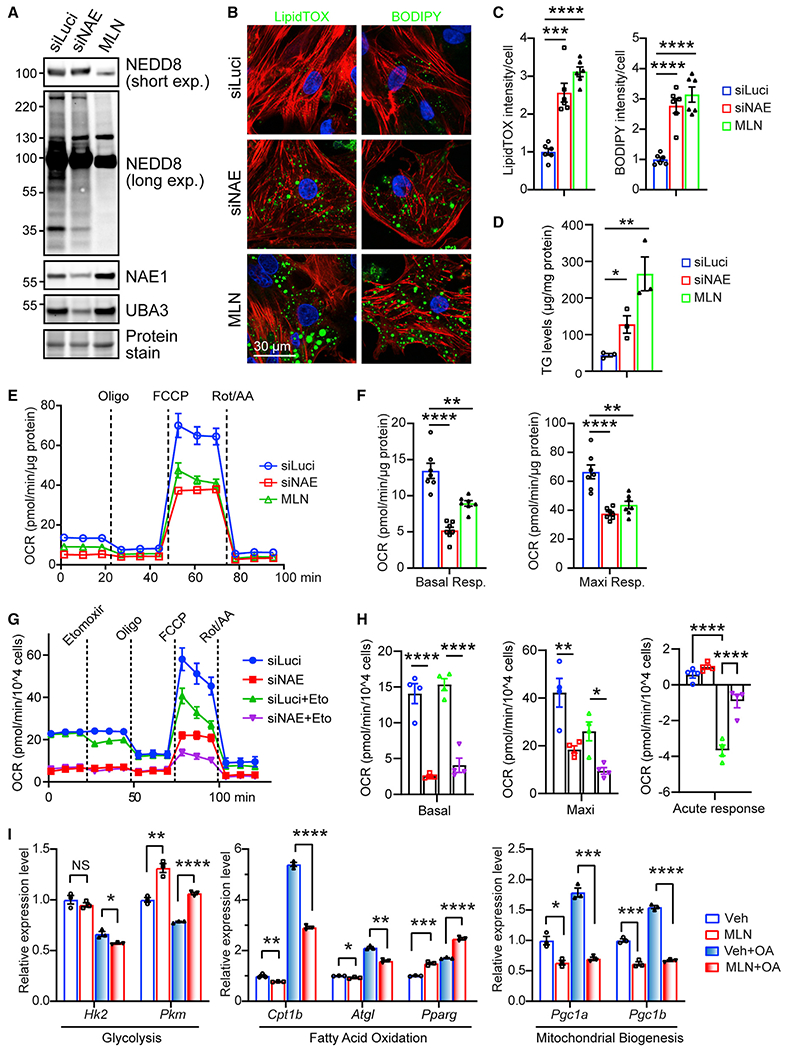
Inhibition of neddylation impairs fatty acid utilization in NRVCs (A–F) NRVCs were treated with siRNA against luciferase (siLuci) or co-transfection with siRNAs against NAE1 and UBA3 (siNAE) or siLuci followed by treatment with MLN4924 (MLN, 1 μM) for 48 h. (A) Western blots of the indicated proteins. NEDD8 blots with short or long exposure (exp.) time are shown. (B) Identification of lipid droplets in NRVCs by LipidTOX (left, green) and BODIPY (right, green) staining. Phalloidin (red) and DAPI (blue) were counterstained to identify cardiomyocytes and nucleus, respectively. Scale bar, 30 μm. (C) Quantification of LipidTOX and BODIPY fluorescence intensity per cell; n = 6 replicates/group. (D) Quantification of triglyceride (TG) levels; n = 3 replicates/group. (E and F) Seahorse analysis of oxygen consumption rate (OCR) (E) and quantification (F) of basal and maximal respiration (Resp.); n = 7 replicates/group. (G and H) Fatty acid oxidation assay by Seahorse analysis of OCR (G) and quantification (H) of basal respiration (Basal), maximal respiration (Maxi), and acute response (OCR_2nd stage_ – OCR_first stage_). Etomoxir (Eto), a CPT1a inhibitor, was used to inhibit fatty acid utilization; n = 4 replicates/group. (I) Quantitative real-time PCR analysis of the indicated genes in NRVCs treated with or without MLN (1 μM) for 48 h and oleic acid (OA; 100 mM) for 24 h; n = 3 replicates/group. *p < 0.05; **p < 0.01; ***p < 0.001; ****p < 0.0001. Error bars indicate SEM. All statistical analyses were performed by one-way ANOVA followed by multiple comparison.

To study whether neddylation regulates fatty acid utilization in a cell-autonomous manner, neonatal rat ventricular CMs (NRVCs) were treated with MLN4924 (MLN), a potent and specific NAE1 inhibitor with minimal effect on Ub or other Ub-like proteins,^48^ or transfected with siRNAs against both subunits (NAE1 and UBA3) of NAE to inhibit neddylation (Figures 5A and S5A). Since cultured CMs are prone to using glucose as a major energy source, NRVCs were treated with oleic acid (OA) to boost fatty acid utilization. Silencing of NAE and MLN treatment led to greater accumulation of lipid as measured by LipidTOX and BODIPY labeling and quantification of triglyceride contents (Figures 5C, 5D, and S5C–S5F). Mitochondrial stress test revealed that both treatments significantly impaired mitochondrial bioenergetics and resulted in significantly diminished basal and maximal respiration (Figures 5E and 5F). Palmitate oxidation stress test further revealed that silencing of NAE suppressed fatty acid utilization (Figures 5G and 5H). MLN treatment significantly blunted OA-induced upregulation of genes crucial for fatty acid utilization, such as *Cpt1b* and *Atgl,* and mitochondrial biogenesis, such as *Pgc1a* and *Pgc1b,* and attenuated OA-induced downregulation of the glycolytic gene *Pkm,* although it had little effect on *Pparg* and *Hk2* expression (Figure 5I). MLN also significantly decreased the expression of PGC1α (a master regulator of mitochondrial biogenesis), CPT1B (carnitine palmitoyltransferase 1B; the rate-controlling enzyme of long-chain fatty acid β-oxidation), and ACSL1 (acyl-CoA synthetase long-chain family member 1; an isozyme of the long-chain fatty acid-coenzyme A ligase) (Figures S5A and S5B). Together, these data suggest that inhibition of neddylation represses fatty acid oxidative metabolism in CMs.

### Metabolomics analysis revealed altered metabolic intermediates in MLN-treated NRVCs

To understand the broader impact of neddylation on CM metabolism, we performed untargeted metabolomics analysis of cultured CMs with and without MLN treatment. Principal-component analysis (PCA) of the detected metabolites clearly distinguished between vehicle (Veh)- and MLN-treated cells (Figure S6A). Among 1,436 annotated metabolites (Table S2), 205 were significantly downregulated, while 208 were upregulated, in MLN-treated CMs (fold change [FC] > 1.5, adjusted p < 0.05; Figure S6B). Integrated pathway enrichment analysis of the significantly altered metabolites identified many metabolic pathways significantly affected by neddylation inhibition (Figure S6C and Table S2). Pathways with top enrichment included critical cellular glucose and fatty acid metabolism, such as fatty acid biosynthesis, carnitine synthesis, Warburg effect, and glycolysis, as well as multiple interconnected amino acid metabolic pathways. Kyoto Encyclopedia of Genes and Genomes (KEGG) pathway analysis of these metabolites also identified similar enriched pathways (Figure S6D). Individual metabolites whose levels were altered by treatment with MLN are shown in Figure S6E. Taken together, the results of the metabolomics analysis suggest that inhibition of neddylation has a global impact on the metabolic profile, especially in the pathways related to glucose and fatty acid metabolism in CMs.

### Persisting HIF1α signaling in neddylation-deficient CMs

**Figure 6.**
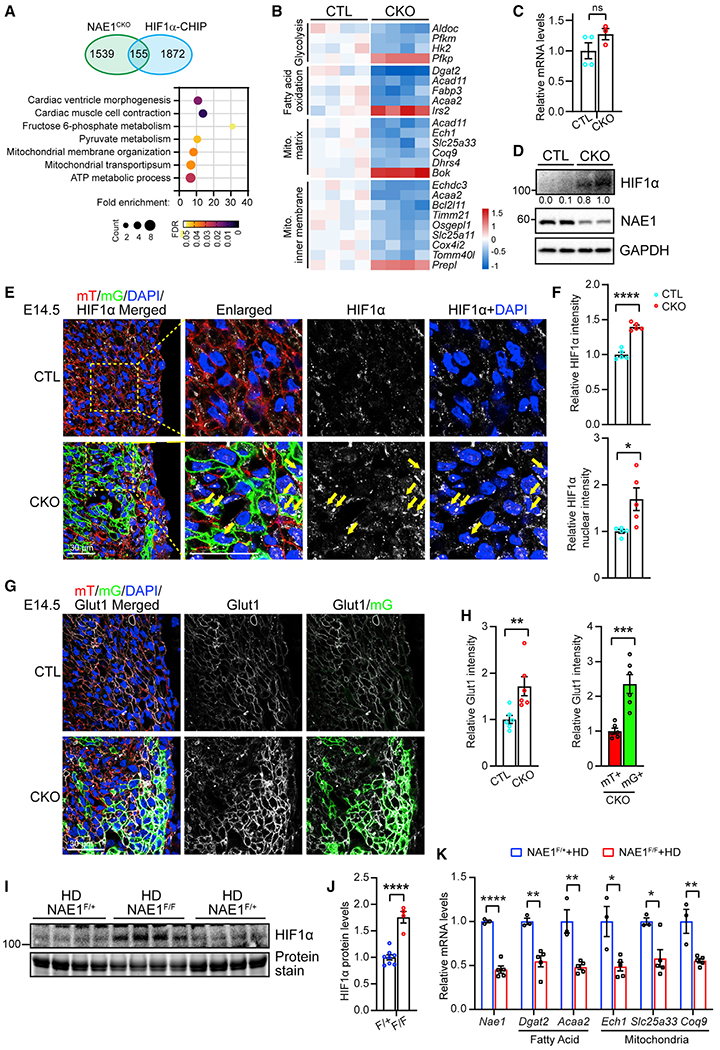
Activation of HIF1α signaling in NAE1-deficient hearts (A) Venn diagram (top) of differentially expressed genes in hearts from NAE1^CKO^ mice and HIF1α ChIP-seq analysis and Gene Ontology analysis (bottom) of the 155 overlapping genes. (B) Heatmap showing the expression levels of selected genes in glycolysis, fatty acid oxidation, and mitochondria among the overlapping genes. (C) Quantitative real-time PCR analysis of HIF1α transcripts in neonatal mouse hearts; n = 4 vs. 3. (D) Western blots of the indicated proteins in neonatal mouse hearts. (E) Confocal images of E14.5 myocardium cryosections immunostained with antibody against HIF1α. Yellow arrows mark nuclei with enriched HIF1α staining. Scale bars, 30 μm. (F) Quantification of relative HIF1α fluorescence intensity and of relative HIF1α fluorescence intensity in the nuclei; n = 5 vs. 5. (G) Confocal images of E14.5 myocardium cryosections immunostained with antibody against Glut1. Scale bar, 30 μm. (H) Quantification of overall relative Glut1 fluorescence intensity in hearts from control (CTL) and NAE1^CKO^ mice, as well as Glut1 fluorescence intensity in mT^+^ CMs and mG^+^ CMs in hearts from NAE1^CKO^ mice; n = 6 vs. 6. (I and J) Western blots (I) and quantification (J) of HIF1α expression in hearts of 4-week-old indicated mice infected with high dose (HD) of AAVs; n = 8 vs. 4. (K) Quantitative real-time PCR analysis of the indicated genes in hearts of 4-week-old mice infected with HD AAVs; n = 3 vs. 5. *p < 0.05; **p < 0.01; ***p < 0.001; ****p < 0.0001; ns, not significant. Error bars indicate SEM. All statistical analyses were performed by one-way ANOVA followed by multiple comparison.

**Figure 7.**
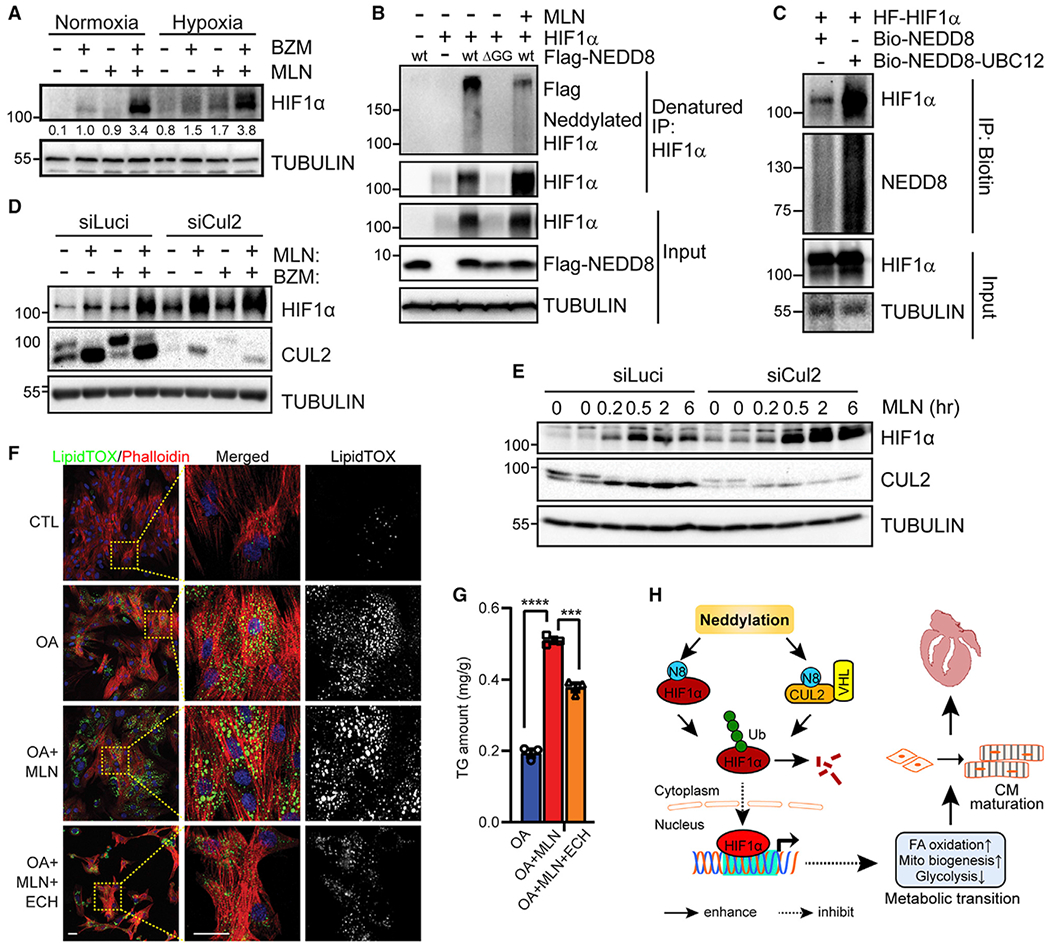
Neddylation of HIF1α regulates its expression (A) Western blot of the indicated proteins in NRVCs treated with bortezomib (BZM; 100 nM) and/or MLN (1 μM) for 24 h under normoxic or hypoxic conditions. Densitometry of HIF1α is shown below the blot. (B) Immunoprecipitation of HIF1α and immunoblotting of the indicated proteins in HEK293 cells transfected with the indicated plasmids. (C) Affinity purification of biotinylated neddylated proteins in HEK293 cells transfected with the indicated plasmids, followed by immunoblotting of the indicated proteins. (D) Western blots of the indicated proteins. NRVCs were transfected with siRNA targeting luciferase control (Luci) or cullin 2 (Cul2) for 48 h, followed by BZM (100 nM) or MLN (1 μM) treatment for an additional 24 h (E) Western blot of the indicated proteins. NRVCs were transfected with siRNA targeting Luci or Cul2 for 48 h and then treated with MLN for the indicated times. (F) Representative images of LipidTOX staining. Scale bars, 50 μm. NRVCs were treated with MLN for 48 h before being incubated with oleic acid (OA; 100 mM) in the presence or absence of echinomycin (ECH) for an additional 24 h. (G) Triglyceride (TG) content in NRVCs treated under the same conditions as in (F); n = 3 replicates/group. (H) A proposed model. Neddylation regulates CM maturation and postnatal cardiac development by mediating metabolic transition in part in an HIF1α-dependent manner. ***p < 0.001; ****p < 0.0001. Error bars indicate SEM. Statistical analysis in (G) was performed by two-way ANOVA followed by multiple comparison.

Activation of HIF1α promotes glycolysis, suppresses fatty acid oxidation, and inhibits mitochondrial biogenesis and oxidative phosphorylation.^49–51^ Developmental downregulation of HIF1α in the heart is essential for perinatal metabolic transition and cardiac chamber development.^6^ The enrichment of dysregulated genes in HIF1α signaling (Figure 3I) prompted us to explore a possible link between neddylation and HIF1α signaling. Analysis of chromatin immunoprecipitation sequencing (ChIP-seq) data of HIF1α in embryonic day 12.5 mouse hearts^21^ showed that 155 (9.1%) of the dysregulated genes in NAE1-deficient hearts were putative HIF1α targets (Figure 6A). Gene ontology (GO) enrichment analysis demonstrated that these 155 genes were mostly associated with fructose and pyruvate metabolism and mitochondria, as well as cardiac muscle contraction and morphogenesis (Figure 6A). Specifically, many HIF1α-regulated glycolytic, fatty acid oxidative, and mitochondrial genes were downregulated in NAE1-deficient hearts (Figure 6B). We confirmed that deletion of NAE1 caused a substantial accumulation of HIF1α proteins without altering its transcripts (Figures 6C and 7D). Consistent with previous reports,^6,21^ HIF1α was abundant in E9.5 mouse hearts but remarkably downregulated in E14.5 mouse hearts and mainly resided in the cytosol of CMs (Figures S7A and 6E). However, HIF1α accumulated in the nucleus of NAE1-deficient CMs at E14.5 (Figures 6E and 6F), a time point when cardiac morphology was comparable between control and mutant hearts.^41^ Moreover, Glut1, a glucose transporter that is a known HIF1α downstream target, was upregulated in mG^+^ (NAE1-deleted) CMs, but not in mT^+^ CMs, in E14.5 mouse hearts (Figures 7G and 7H), supporting the activation of HIF1α. Similarly, depletion of NAE1 via AAV-Cre also led to accumulation of HIF1α in postnatal hearts (Figures 6I and 6J) and downregulation of HIF1α target genes involved in fatty acid metabolism *(Dgat2, Acaa2)* and mitochondrial function (*Ech1, Slc25a33, Coq9*) (Figure 6K). HIF1α reporter assay showed that both silencing of NAE and MLN treatment increased HIF1α activity in H9C2 and HEK293 cells (Figures S7B–S7D). Taken together, these results suggest that inhibition of neddylation promotes accumulation and activation of HIF1a, which in turn represses the establishment of oxidative metabolism in the developing heart.

### Neddylation regulates HIF1α expression in Cul2-dependent and -independent manners

HIF1α expression is largely controlled at the posttranscriptional level. Under normoxic conditions, HIF1α is ubiquitinated by Cul2-von Hippel-Lindau (VHL) ubiquitin ligase and subsequently degraded by the proteasome.^52^ Neddylation is essential for cullin-RING ubiquitin ligase activity,^53^ and inhibition of neddylation via MLN or NAE1^CKO^ robustly increased HIF1α in CMs (Figures 6D, 6I, 7A, and S7E). Interestingly, however, MLN and bortezomib (BZM; a 20S proteasome inhibitor) had synergistic effects in stabilizing HIF1α in CMs under both normoxic and hypoxic conditions (Figure 7A), suggesting that neddylation functions independent of Cul2 to control HIF1α expression. Immunoprecipitation of HIF1α under denatured conditions, which prevents non-covalent protein-protein interactions, identified neddylated species, which were absent in cells expressing conjugation-deficient NEDD8 mutant and reduced by MLN treatment (Figure 7B). Moreover, overexpression of NEDD8 E2 enzyme UBC12 also increased neddylated HIF1α (Figure 7C), further supporting HIF1α as a putative NEDD8 target. Furthermore, MLN remained effective in stabilizing HIF1α in Cul2-deficient CMs (Figures 7D and 7E). These data suggest that neddylation of HIF1α promotes its degradation in a manner that is independent of Cul2 ubiquitin ligase. Inhibition of HIF1α with echinomycin (Ech), a compound that inhibits HIF1α binding to DNA and thus its transcriptional activity,^54^ substantially reduced MLN-induced lipid accumulation in CMs (Figure 7F), and this was further confirmed by quantification of triglyceride (TG) content (Figure 7G). Thus, we propose that neddylation has dual roles in the regulation of HIF1α expression and that upregulation of HIF1α affects fatty acid utilization in neddylation-deficient CMs.

## DISCUSSION

In this study, we demonstrated that neddylation is required for CM maturation and perinatal cardiac development by ensuring developmental metabolic transition. Our findings support a model in which neddylation modifies HIF1α and Cul2, and neddylation of both proteins has a synergistic effect in promoting efficient HIF1α ubiquitination and degradation, which is essential for the establishment of oxidative metabolism in the developing heart (Figure 7H). Consequently, inhibition of neddylation leads to accumulation of HIF1α proteins and persistent HIF1α signaling, which represses fatty acid utilization in late gestational and postnatal heart, leading to defects in CM maturation and eventually heart failure. Thus, our study identifies neddylation as a crucial posttranslational mechanism regulating cardiac maturation and metabolism.

The biological functions of neddylation have been primarily described in the context of its impact on cell differentiation and proliferation,^31–35^ but its significance in terminally differentiated, postmitotic organs has not been investigated. Using AAV-TnT-Cre to achieve mosaic, postnatal gene deletion in the heart, our study defines a role for neddylation in CM maturation. This is evidenced by a disrupted T-tubule network, decreased CM cell size, and significantly dysregulated expression of maturation genes in neddylation-deficient CMs in the absence of overt cardiac dysfunction (Figures 2 and 3). Consistent with an arrest in cardiac maturation, mice with high-level deficiency of neddylation developed cardiomyopathy and heart failure before adolescence (Figure 1), and genetic deletion of NAE1 in embryonic heart at midgestational stage via αMHC^Cre^ induces heart failure and perinatal lethality.^41^ Notably, deletion of CSN8, a subunit of the deneddylase COP9 signalosome (CSN), in postnatal hearts via αMHC^Cre^ causes dilated cardiomyopathy and heart failure by 3 weeks of age.^38^ Transient inhibition of neddylation with MLN4924 during the first week after birth predisposes the heart to isoproterenol-induced pathological remodeling.^55^ Since perturbations of deneddylation or neddylation in these studies occurred in the critical time window of CM maturation, disruption of CM maturation may contribute to the observed cardiac phenotypes. ASB2, a Cul5-RING Ub ligase whose activity is regulated by neddylation, was shown to regulate CM maturation by facilitating sarcomere organization and formation of cell-cell junctions.^56^ Together, recent findings from our studies and others support a previously unrecognized mechanism regulating CM maturation and perinatal cardiac development.

Increasing evidence has suggested a critical role for a metabolic shift from glycolysis to oxidative metabolism in CM maturation. Perturbations of PGC1/PPAR signaling, an important pathway regulating mitochondrial biogenesis and maturation, disrupts CM maturation, whereas PGC1/PPAR activation improves the maturation of pluripotent stem cell-derived CMs.^5^ Similarly, disruption of ERRα and ERRγ, which function as critical transcriptional activators of metabolic genes in adult CM, impairs cardiac maturation and results in heart failure in the developing heart.^19^ Findings from this study establish neddylation as a crucial mechanism in the regulation of cardiac oxidative metabolism. We present *in vitro* and *in vivo* evidence showing that inhibition of neddylation disrupts the expression of metabolic genes, inhibits mitochondrial maturation and respiration, suppresses fatty acid utilization, and significantly alters metabolite profiles in CMs (Figures 3, 4, 5, and S3–S6), which collectively culminate in the arrest of CM maturation. Consistent with our results, inhibition of neddylation via deletion of NEDD8 or UBA3 in the liver suppressed mitochondrial oxidative phosphorylation and fatty acid oxidation, leading to hepatic steatosis.^33^ Interestingly, MLN4924 treatment and UBA3 knockdown were shown to enhance basal and maximal oxidative phosphorylation in cancer cell lines,^57^ suggesting that the effect of neddylation in metabolism could be cell-type dependent.

The spatiotemporal downregulation of HIF1α in the developing heart is crucial for the metabolic transition from glycolysis to oxidative metabolism and cardiac maturation. Despite its essential role in stimulating CM proliferation in hypoxic embryonic hearts,^21^ persistent HIF1α activation in postnatal hearts has been linked to heart failure and premature death, at least in part due to defective energy metabolism.^17,58–61^ Deletion of the E3 ubiquitin ligase VHL in the developing heart results in HIF1α hyperactivation, abrogating the developmental metabolic shift and impairing cardiac maturation and function, which can be rescued by concomitant deletion of HIF1α.^6^ Moreover, chronic perinatal hypoxia is sufficient to delay cardiac maturation.^62^ We observed accumulation of HIF1α (Figures 6 and 7) and deficits in oxidative metabolism (Figures 4 and 5) in neddylation-deficient hearts and cultured CMs, and inhibition of HIF1α attenuated lipid accumulation in neddylation-deficient CMs (Figure 7G). Thus, our data suggest that neddylation regulates developmental metabolic reprogramming, at least in part, by repressing HIF1α signaling the heart.

Identification of NEDD8 substrates is of utmost importance to elucidate the biological functions of neddylation. Consistent with the findings in non-cardiac cells,^63–66^ we confirmed that HIF1α is degraded by Cul2-VHL Ub ligase and is also a putative NEDD8 target in CMs (Figure 7). Interestingly, inhibition of neddylation in the absence of Cul2 remains effective in stabilizing HIF1α, suggesting that neddylation of HIF1α promotes its degradation. Thus, neddylation appears to regulate HIF1α by directly targeting Cul2 and HIF1α, respectively. While neddylation itself does not directly target the modified substrates for proteasomal degradation under basal conditions, it is reported that NEDD8 incorporates into the existing Ub chain under stress conditions to facilitate the degradation of ubiquitinated proteins,^67,68^ which may otherwise prevent the exhaustion of the ubiquitin machinery. Further experiments are needed to identify the neddylation site on HIF1α and elucidate the biological functions of HIF1α neddylation.

As a protein modification that targets diverse cellular proteins,^69,70^ neddylation may have multiple downstream effectors that coordinate CM metabolism. Other than HIF1α, mitochondrial electron transfer flavoproteins A and B (ETFA and ETFB), two key proteins responsible for relaying electrons in the electron transport chain, were identified as neddylated substrates, and neddylation increased the stability of ETFA and ETFB by preventing their ubiquitination and degradation in hepatocytes.^33^ PPARγ neddylation was reported to control the expression of genes involved in fatty acid storage and is essential for adipogenesis.^32^ Whether neddylation of these proteins regulates cardiac developmental metabolic reprogramming remains to be determined. It should be pointed out that the NEDD8 proteome is likely cell-type specific. Mapping the NEDD8 proteome landscape will provide mechanistic insights into how neddylation regulates cardiac homeostasis.

### Limitations of the study

There are still some limitations in this study. First, we cannot exclude the possibility that neddylation inhibition perturbs the Hippo-YAP pathway, as we reported previously,^41^ which may have effects on CM metabolism^71^ and the last round of proliferation and division.^72^ Second, single-cell or single-nucleus RNA-seq of cells isolated from hearts infected with medium- or low-dose AAV-Cre will provide precise transcriptomic changes in neddylation-deficient CMs, which may define the metabolic phenotype independent of ongoing cardiac remodeling. Third, neddylation may have a direct impact on multiple pivotal cellular processes beyond cardiac metabolism and maturation, such as CM contractility, T-tubule remodeling, and Ca^2+^ handling. Defects in these pathways may contribute to the cardiac phenotype observed in NAE1-deficient hearts.

## STAR★METHODS

### RESOURCE AVAILABILITY

#### Lead contact

Further information and requests for resources and reagents should be directed to and will be fulfilled by the lead contact, Huabo Su (hsu@augusta.edu).

#### Materials availability

All reagents generated in this study will be made available on request from the lead contact with a completed Materials Transfer Agreement.

#### Data and code availability

- All sequencing data have been deposited at GEO and are publicly available as of the date of publication. The accession number is listed in the key resources table (GEO: GSE217964). All microscopy data in this paper will be available upon request to the lead contact.
- No specific code was used in this article.
- Any additional information required to reanalyze the data reported in this paper is available from the lead contact upon request.

### EXPERIMENTAL MODEL AND SUBJECT DETAILS

#### Animals

All animal experiments were approved by the Augusta University Institutional Animal Care and Use Committee. A transgenic mouse line bearing a NAE1^Flox^ allele was crossed with αMHC^Cre^ mice (the Jackson Laboratory, strain # 011038) to generate CM-restricted NAE1 knockout (NAE1^CKO^) mice. A transgenic mouse line bearing the Rosa26^mTmG^ allele (the Jackson Laboratory, strain # 007676) was used for lineage tracing. These mice were maintained in the C57BL/6J inbred background for our studies.

During all experiments involving transgenic mice, experimental mice were randomly assigned into all groups, including sex (male and female), age (8–12 weeks for breeding/4, 6 weeks or neonatal P1 as specified in each figure), unless specified. The total estimate number of mice used in this article is ~180 mice (including embryonic stage mice) and ~120 rat pups for NRVC isolation. All mice were at healthy status before experiments. No subjects were involved in previous procedures except for AAV injections indicated in specific figures at age of P1. The housing conditions were well maintained at room temperature in the animal facility of Augusta University. The influence of sex was not determined for 4 or 6 weeks sacrificed mice. The influence of sex of P1 pups was considered as not significant.

#### Ethical statement

Our studies did not include human participants, human data, or human tissue. All animal protocols were approved by the Institutional Animal Care and Use Committee (IACUC) at Augusta University.

#### Culture of neonatal cardiomyocytes and cell lines

Neonatal rat ventricular cardiomyocytes (NRVCs) were isolated using Neonatal Cardiomyocyte Isolation System (Worthington) following the manufacturer’s protocol.^40^ Briefly, neonatal rat hearts were minced into ~1 mm diameter and digested in 0.05% Trypsin in 4°C overnight on a slow speed rocker. The other day, digested heart tissue was washed 4 times and subject to Collagenase digestion at 37°C for 40 min. NRVCs were next separated by pipet flushing and through 70 mm filter, pre-plated for 2 h to exclude cardiac fibroblast, and finally plated in 60-mm dishes in DMEM containing 10% FBS, 1% P/S and 1x BrdU for two days. The culture media was next changed to 2% FBS, which could be cultured in 37°C incubator with 5% CO_2_ for at most 3 days prior to use. Hypoxia condition was created by N_2_ humidified chamber with 5% CO_2_ and 1% O_2_ supplied at 37°C for 24 h.

HEK293 cells were cultured in DMEM supplemented with 10% fetal bovine serum at 37°Cand 5% CO_2_. pShuttle-CMV-SF-NEDD8 (Strep- and FLAG-tagged NEDD8) was generated as described.^40^ The plasmids were transfected into the cells using X-tremeGene HP DNA transfection reagent (Roche) according to the manufacturer’s protocol. Cells were harvested for analysis 48-72 h after transfection or as indicated.

Some of the cells were treated with vehicle (DMSO or BSA), 1 μM MLN4924 (Active Biochem), 100 nM bortezomib (Enzolife), or 1 nM Echinomycin (Thermo Fisher) where applicable.

### METHOD DETAILS

#### Adeno-associated virus infection

AAV9 virus expressing Cre under the control of a mouse cardiac troponin T promoter (AAV9-cTnT-Cre)^42^ (Vigene Biosciences) was subcutaneously injected into mice at age P1 at the indicated doses (high dose: 5 x 10^11^ GC/pup; medium dose: 0.5 × 10^11^ GC/pup; low dose: 0.25 x 10^11^ GC/pup). Genotyping of mouse pups were blind to injection performer.

#### *In situ* t-tubule labeling, imaging, and quantification

The hearts from AAV-infected mice were isolated and perfused with FM4-64FX and subjected to *in situ* confocal microscopy. The t-tubule content, integrity and regularity were next analyzed in ImageJ following previously described STAR Methods.^79^ Briefly, intact mouse hearts were Langendorff-perfused at room temperature with Tyrode’s solution (NaCl 137, KCl 5.4, HEPES 10, Glucose 10, MgCl_2_ 1, NaH_2_PO_4_ 0.33, pH adjusted to 7.4 with NaOH, oxygenated with 95% O_2_ and 5% CO_2_ during experiments), containing 10 μM FM4-64FX, a lipophilic fluorescence indicator of membrane structure (Thermo Fisher), for 20 min. The membrane structure of epicardial myocytes was analyzed *in situ* with confocal microscope (STELLARIS 8, Leica Microsystems). T-tubule images were next analyzed with IDL image analysis program (ITT VIS Inc., Colorado). Background noise in confocal images was filtered with a threshold value retrieved from image intensity histogram. T-tubule two-dimensional images were converted to frequency domain using the Fast Fourier Transformation function in IDL, so that it could be determined whether repeating patterns occur (T-tubule regularity) and how strong the repeating patterns are (T-tubule power).

#### RNA-seq analysis

Mouse ventricles were minced and treated with RNAlater (Thermo Fisher) according to the manufacturer’s protocol at −80°C. The isolated RNA was subjected to RNA-sequencing analysis performed by Genome Technology Access Center (GTAC) at Washington University via Next Generation Sequencing. Genotyping information of the mice was pre-coded and blind during RNA-seq analysis and data analysis.

#### Lipid droplets staining assays

Cells were stained with LipidTOX kit or BODIPY staining following the manufacturer’s protocol (Thermo Fisher #H34475 or #D3922, respectively). Briefly, after indicated treatment, NRVCs grown on coverslips were fixed in 4% paraformaldehyde at room temperature for 10 min and subjected to LipidTOX labeling (1: 1000 in PBS, RT for 30 min) or BODIPY labeling (1: 5000 in PBS, RT for 30 min). The cells were counterstained with cardiac Troponin-T (TnT)/Phalloidin (Thermo Fisher) and DAPI (diamidino-2-phenylindole, Thermo Fisher) to label cardiomyocytes and nuclei, respectively. Confocal images were quantified by ImageJ. LipidTOX/BODIPY intensity was normalized with DAPI intensity. Eight views/sample, 3 samples/group were quantified.

#### Triglyceride (TG) measurement

Cells were lysed in 1% Triton X-100 in PBS. The concentrations of TG were measured using a TG assay kit (Infinity Triglycerides kit) following the manufacturer’s protocol. The colormetric readings were collected with a microplate reader at OD 570 nm and the data were normalized to total proteins.

#### Metabolomics analysis

Cell pellets (~2.4X10^7^ cells/biological replicate) were collected and snap frozen in liquid nitrogen for untargeted metabolomics analysis (Creative Proteomics, NY). A total of 4 control samples and 4 MLN-treated samples were included in this analysis. No sample was excluded since sample submission. Briefly, samples were thawed in 800 μL of 80% methanol, sonicated at 4°C for 30 min, kept at −40°C for 1 h, vortexed for 30 s, and centrifuged at 12000 rpm at 4°C for 15 min. Finally, 200 μL of supernatant and 5 μL of DL-o-chlorophenylalanine (140 μg/mL) were used for LC-MS analysis. Quality control (QC) samples were prepared using the same sample preparation procedure. Samples were separated by Ultimate 3000LC combined with Q Exactive MS (Thermo) and screened with ESI-MS. The LC system is comprised of an ACQUITY UPLC HSS T3 (100 × 2.1 mm × 1.8 μm) with Ultimate 3000LC. The mobile phase was composed of solvent A (0.05% formic acid water) and solvent B (acetonitrile) with a gradient elution. The flow rate of the mobile phase was 0.3 mL/min. The column temperature was maintained at 40°C, and the sample manager temperature was set at 4°C. Mass spectrometry parameters in ESI- mode are as follows: heater temperature 300°C, sheath gas flow rate, 45 arb; auxiliary gas flow rate, 15arb; sweep gas flow rate, 1 arb; spray voltage, 3.2 kV; capillary temperature,350°C; S-Lens radiofrequency level, 60%. Sample information of specific treatments was blind during metabolomics analysis and data collection.

The raw data were acquired and aligned using MetaboAnalyst 5.0^79^ based on the m/z value and the retention time of the ion signals. Principal component analysis, statistical analysis, enrichment analysis, pathway analysis and networking analysis were also performed under the MetaboAnalyst 5.0 platform. A value of p < 0.05 was considered statistically significant unless specifically defined.

#### Embryo isolation

Female mice crossed with their mating partners were checked for plug formation. Mouse embryos at different embryonic ages were dissected and washed in cold PBS. Embryo heads and lower dorsal parts were removed prior to fixation. For immunostaining, the embryos were fixed in cold 4% paraformaldehyde overnight at 4°C, 70% ethanol overnight at 4°C, and subjected to either OCT embedding for cryosection or paraffin processing and embedding for paraffin sections at thickness of 5 μm. Approximately 60 embryos within 8 litters were used in this article. The grouping was determined by the genotyping of yolk sac of individual embryo. Sex was not considered at this stage of embryonic development.

#### Echocardiography

Perinatal mice were anesthetized by inhalation of isoflurane (2.5% for induction and 1.5% for maintenance) via a nose cone. The adequacy of anesthesia was monitored by toe pinch. Cardiac images and loops were recorded using a VEVO 2100 echocardiography system with a 30MHz transducer (Visual Sonics). The LV morphometric and functional parameters were analyzed offline using VEVO 2100 software. Echocardiography of conscious neonates was performed by gently securing the mice on the station with tapes. Mice information including genotyping and treatment was blind to echocardiography performer.

#### Histology and immunohistochemistry analysis

For histology analysis, 5-μm OCT-embedded cryosections were subjected to Fast Green/Sirius Red staining. For immunohistochemistry analysis, cryosections were subjected to antigen retrieval in preheated sodium citrate buffer (pH 6.0, 98°C) for 10 min with the PT Link system (Dako). For cryo-sectioned tissues, deparaffinizing and antigen retrieval procedure were replaced by treatment of 1% Triton X-100 in PBS for 10 min at room temperature. After pre-incubation with 10% non-immune goat serum (Thermo Fisher Scientific) to prevent non-specific binding, tissue sections were incubated with primary antibodies at 4°C overnight and subsequently with appropriate Alexa-Fluor conjugated secondary antibodies (Thermo Fisher Scientific) for 1 h at room temperature. Finally, sections were stained with DAPI (Sigma) and mounted in VECTASHIELD antifade mounting medium (Vector Laboratories). Images were captured with Olympus BX41 (Olympus) or Zeiss Upright 780 confocal microscope (Zeiss). Section information was pre-coded and blind to procedure performers including embedding, sectioning, staining and imaging.

#### Protein extraction and Western blot analysis

Protein was extracted from ventricular myocardium tissues or cultured cells, concentration determined with BCA reagents (Thermo Fisher Scientific), and SDS-PAGE, immunoblotting, and densitometry analysis were performed as previously described.^38^ Briefly, frozen tissue or cell was homogenized in lysis buffer (50 mm Tris-HCl pH 6.8 containing 1% SDS, 10% glycerol, and complete protease inhibitor mixture), sonicated and spin down at 14,000 rpm. The supernatant was collected and boiled for 10 min. After protein concentration was determined, the protein lysate was mixed with half the volume of 3x SDS loading buffer with 15% β-mercaptoethanol. The mixed sample was next boiled for 5 min and subjected to SDS-PAGE gel isolation, transferred to PVDF membrane, and blotted with specific primary antibody followed by secondary antibody conjugated with HRP and film development. No unique technique of Western blot was conducted. Primary antibodies used in this study are listed in key resources table and detailed in Table S3.

All uncropped WB figures can be found in Supplemental Materials.

#### Immunoprecipitation

For denaturing IP, cells were lysed in TSD buffer (50 mM Tris pH 7.5, 1% SDS, 5 mM DTT) containing a cocktail of phosphatase and protease inhibitors (Sigma). The lysates were then diluted with 10-fold volume of TNN buffer (50 mM Tris pH 7.5, 250 mM NaCl, 5 mM EDTA, 0.5% NP-40), and incubated with primary antibody and protein A Sepharose beads (ThermoFisher Scientific) with rotation at 4°C overnight. For Biotin-IP, cells were lysed by RIPA buffer (NaCl 150mM, Tris-HCl pH 7.4 10mM, EDTA 1mM, Triton X-100 1%, SDS 0.1%, Sodium deoxycholate 0.1%) and incubated with NeutrAvidin agarose resin (Thermo Scientific, #29201) with rotation at 4°C overnight. Immunoprecipitates were eluted with SDS-PAGE sampling buffer at 95°C for 5 min, and subjected to SDS-PAGE and immunoblotting.

#### RNA preparation and real-time PCR

Isolation of total RNA and reverse transcription into single-stranded cDNA was performed as previously described.^41^ Briefly, Total RNA was isolated from heart tissue or NRVCs using the TRIzol Reagent (Invitrogen) following the manufacturer’s protocol. Gene expression levels were measured in at least triplicate per sample by real-time quantitative PCR (StepOnePlus Real-Time PCR system, Thermo Fisher Scientific) using the SYBR-Green assay with gene-specific primers at a final concentration of 200 nM. Relative gene expression was calculated using the 2^−ΔΔct^ method against a rat house-keeping gene acidic ribosomal phosphoprotein P0 (RPLP0) or a mouse house-keeping gene hypoxanthine guanine phosphoribosyl transferase 1 (Hprt) as appropriate. The primers used for qPCR are listed in Key Resources Table and detailed in Table S4.

#### siRNA transfection

siRNAs against rat *Cul2* (5’ – TTCGAGCGACCAGTAACCTTA-3′) and luciferase (5’ -AACGTACGCGGAATACTTCGA-3′) were used. Briefly, NRVCs were transfected with siRNAs (100 pmol per 2×10^6^ cells) using Lipofectamine RNAimax (Thermo Fisher Scientific) following the manufacturer’s protocol at 24–48 h after plating. Six hours after the transfection, the siRNA-containing medium was replaced with fresh medium containing 2% FBS. In some experiments, a second round of siRNA transfection may be performed 2 days after the first transfection to achieve sustained gene silencing.

#### ATP content measurement

Cells were lysed in cold 10% trichloroacetic acid (TCA) and diluted 100-fold in Tris-acetate (pH 7.8). ATP content was measured by ATP Bioluminescent Assay Kit (Sigma, #FL-AA) on a luminescent microplate reader and normalized to total protein levels.

#### Dual luciferase assay

The dual luciferase assay was performed following the manufacture protocol of Dual-Luciferase Reporter Assay System (Promega). Specifically, HRE-firefly-luciferase and Renilla luciferase were co-transfected in H9C2 cells and cultured for 72 h. Next, the firefly and Renilla luciferase activities of the same sample were sequentially measured by a luminometer after addition of specific substrates, with the ratio of firefly to Renilla luciferase activity (Fluc/Rluc) as readout of HIF-1α activity.

#### Plasmids

HA-HIF1α (#18949), HRE-firefly-luciferase (#26731), Renilla luciferase (#118016), 5HRE-GFP (#46926) plasmids were obtained from Addgene. Received bacteria were amplified and correlating plasmids were extracted with Plasmid Maxi kit (Qiagen) following manufactures instructions. pShuttle-CMV-FLAG-NEDD8 and pShuttle-CMV-FLAG-NEDD8-dGG plasmids were generated as previously described.^41^ Briefly, NEDD8 or NEDD8-dGG sequence was synthesized and cloned in pShuttle-CMV-FLAG construct. CAGΔX-bioNEDD8-BirAOV5-g2A-puro and CAGΔX -bioNEDD8-BirAOV5-g2A -UBC12 plasmids were a kind gift from James Sutherland at CIC bioGUNE.^77^

### QUANTIFICATION AND STATISTICAL ANALYSIS

Results are shown as mean ± SEM. Paired data were evaluated using a two-tailed Student’s *t*-test. For multiple comparisons, one-way analysis of variance (ANOVA) or when appropriate, two-way ANOVA, followed by post hoc test was performed.

A p-value < 0.05 was considered statistically significant. In figures, different p value was labeled as follow: NS, p > 0.05; *, 0.05 > p ≥ 0.01; **, 0.01 > p ≥ 0.001, ***, 0.001 > p ≥ 0.0001; ****, p < 0.0001. Specific statistical analysis method and n number of individual experiment can be found in related figure legend. All centers of bar graph in this article represent mean value of that group. All error bars in this article represent ±SEM unless stated otherwise in figure legends.

## Supplementary Material

1

2

3

4

5

## Figures and Tables

**Table:** KEY RESOURCES TABLE

REAGENT or RESOURCE	SOURCE	IDENTIFIER
Antibodies		

NAE1	Cell Signaling Technology	Cat# 14321
NEDD8	Cell Signaling Technology	Cat#2754
CUL2	Thermo Fisher Scientific	Cat# 51-1800
MYH6	Proteintech	Cat# 2281-1-Ap
MYH7	Proteintech	Cat# 22280-1-ap
MYL2	Proteintech	Cat# 10906-1-AP
MYL7	Proteintech	Cat#17283-1-AP
Kir2.1	Thermo Fisher Scientific	Cat# MA5-27681
HCN4	Thermo Fisher Scientific	Cat#MA3-903
ACSL1	Thermo Fisher Scientific	Cat# PA5-17136

Bacterial and virus strains		

pEN.AAV9-cTnt-Cre-WPRE	Vigene Biosciences	AAV9SP(VB210825-1182agk)-C

Chemicals, peptides, and recombinant proteins		

Chemical: MLN4924	Active Biochem	N/A
Chemical: bortezomib	Enzo Life Science	N/A
Chemical: Echinomycin	Sigma-Aldrich	Cat# SML0477

Critical commercial assays		

ATP Bioluminescent Assay Kit	Sigma	Cat# FL-AA
Lipofectamine RNAimax	Thermo Fisher Scientific	Cat# 13778150
LipidTOX kit	Thermo Fisher Scientific	Cat# H34475
Infinity Triglycerides kit	Thermo Fisher Scientific	Cat# TR22421
Dual-Luciferase^®^ Reporter Assay System	Promega	Cat# E1910
Plasmid Maxi kit	Qiagen	Cat# 12162
TRIzol RNA isolation kit	Invitrogen	Cat# 15596026

Deposited data		

NAE1CKO RNA-seq analysis	Gene Expression Ominibus	GSE217964

Experimental models: Cell lines		

HEK293 cell line	ATCC	Cat# CRL-1573

Experimental models: Organisms/strains		

Transgenic mouse line: NAE1^Flox^	Previously generated by ourselves^41^	N/A
Transgenic mouse line: αMHC^Cre^	Jackson Laboratory	Cat# 011038
Transgenic mouse line: Rosa26^mTmG^	Jackson Laboratory	Cat# 007676

Oligonucleotides		

siRNAs against rat Cul2 (5’ – TTCGAGCGACCAGTAACCTTA-3’)	Qiagen	Cat# SI01787443
siRNAs against luciferase (5’ -AACGTACGCGGAATACTTCGA-3’)	Qiagen^41^	N/A
qPCR primer: Nae1, Sense, “CAACTCAGATCCCAAGCAGTAT”	Integrated DNA Technologies	N/A
qPCR primer: Nae1, Anti-sense, “CCTTTAAGGCACGAGCTAGAA”	Integrated DNA Technologies	N/A
qPCR primer: Serca2a, Sense, “CCTCCACTTCCTCATCCTCTAT ”	Integrated DNA Technologies	N/A
qPCR primer: Serca2a, Anti-sense, “GATGACTGGCAGTGAGATCTTG ”	Integrated DNA Technologies	N/A
qPCR primer: Nppa, Sense, “CACAGATCTGATGGATTTCAAGA”	Integrated DNA Technologies	N/A
qPCR primer: Nppa, Anti-sense, “CCTCATCTTCTACCGGCATC”	Integrated DNA Technologies	N/A
qPCR primer: Nppb, Sense, “GTCAGTCGTTTGGGCTGTAAC”	Integrated DNA Technologies	N/A
qPCR primer: Nppb, Anti-sense, “AGACCCAGGCAGAGTCAGAA”	Integrated DNA Technologies	N/A

Recombinant DNA		

HA-HIF1α plasmid	Kondo et al.^73^	Addgene, Cat #18949
HRE-luciferase	Emerling et al.^74^	Addgene, Cat #26731
Renilla luciferase	Giacomelli et al.^75^	Addgene, Cat #118016
5HRE-GFP	Vordermark et al.^76^	Addgene, Cat #46926
pShuttle-CMV-FLAG-NEDD8	Previously generated by us(Zou et al.^41^)	N/A
pShuttle-CMV-FLAG-NEDD8-dGG	Previously generated by us^41^	N/A
CAGΔX-bioNEDD8-BirAOV5-g2A-puro	kind gift from James Sutherland at CIC bioGUNE^77^	N/A
CAGΔX -bioNEDD8-BirAOV5-g2A -UBC12	kind gift from James Sutherland at CIC bioGUNE^77^	N/A

Software and algorithms		

MetaboAnalyst 5.0 platform	Public available by Xia Lab^78^	N/A
Microsoft Office	Microsoft	N/A
Photoshop	Adobe	N/A
Illustrator	Adobe	N/A
Endnote 20	Clarivate	N/A
GraphPad Prism 9	Dotmatics	N/A

## Highlights

- Inhibition of neddylation in the developing heart causes heart failure
- Neddylation is required for cardiomyocyte maturation
- Neddylation regulates cardiomyocyte perinatal metabolic reprogramming
- Neddylation targets HIF1α and promotes fatty acid utilization in cardiomyocytes

## INCLUSION AND DIVERSITY

One or more of the authors of this paper self-identifies as an underrepresented ethnic minority in their field of research or within their geographical location. We worked to ensure sex balance in the selection of non-human subjects. We avoided “helicopter science” practices by including the participating local contributors from the region where we conducted the research as authors on the paper. We support inclusive, diverse, and equitable conduct of research.
