# Supplementary material for: Neddylation is required for perinatal cardiac development through stimulation of metabolic maturation
Source: Cell Rep. Author manuscript; Available in PMC 2023 Mar 21. (PMC10029150; doi:10.1016/j.celrep.2023.112018)
Supplement: 1 [file NIHMS1870551-supplement-1.pdf]

Cell Reports, Volume *42*

Supplemental information

Neddylation is required for perinatal cardiac development through stimulation of metabolic maturation

Jianqiu Zou, Wenjuan Wang, Yi Lu, Juan Ayala, Kunzhe Dong, Hongyi Zhou, Jinxi Wang, Weiqin Chen, Neal L. Weintraub, Jiliang Zhou, Jie Li, and Huabo Su

I. Supplemental Figures and supplemental figure legends

Supplemental Figure 1

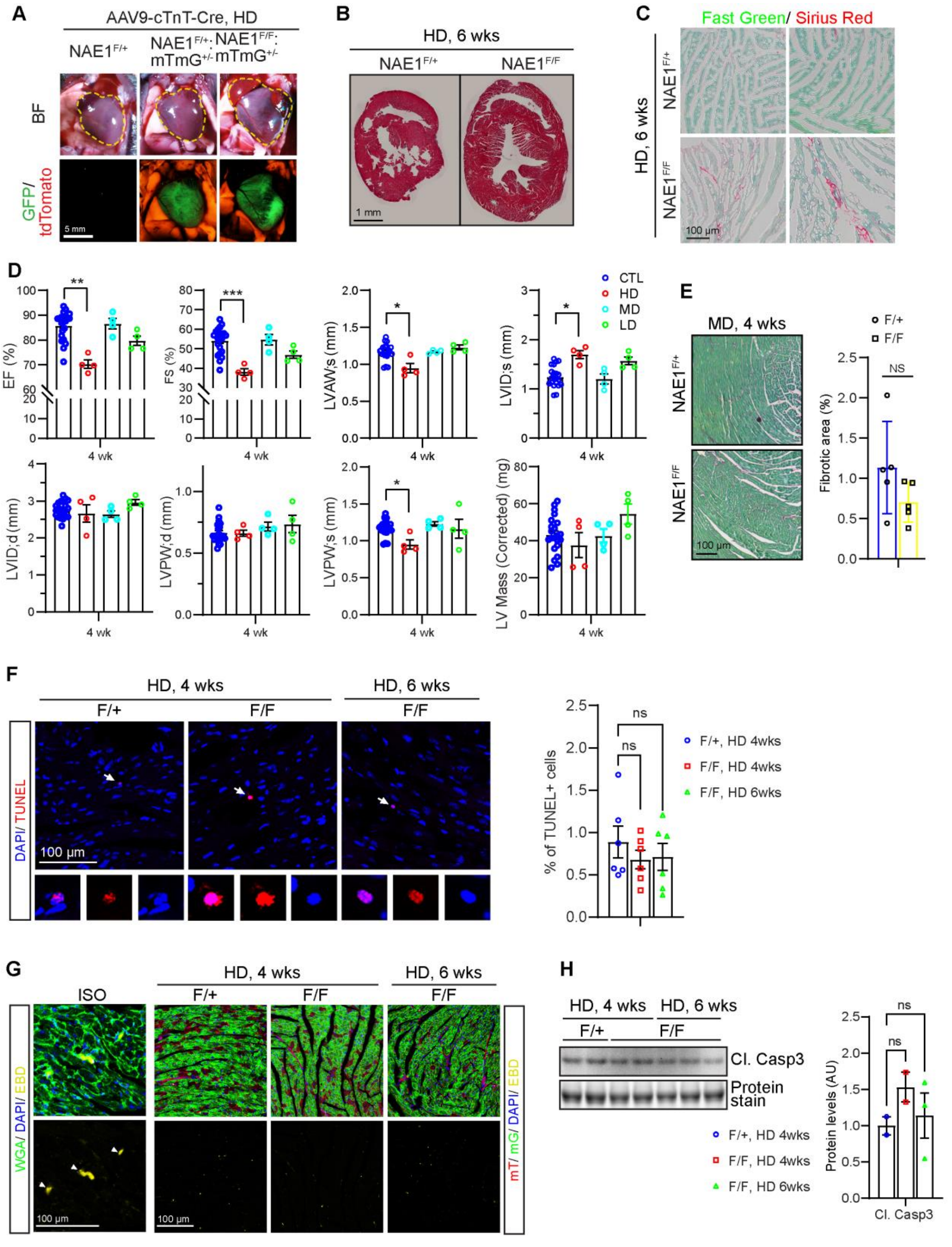

Supplemental Figure 1. Deletion of NAE1 in neonatal hearts results in cardiac dysfunction with negligible impact on cardiomyocyte cell death, Related to Figure 1. A-B, *In situ* bright field and fluorescent images (**A**, Scale bars, 5 mm.) and H&E staining of cross-section (**B**, Scale bars, 1 mm.) of mouse hearts at 6 weeks post high dose (HD) AAV-Cre infection. **C,** Fast Green and Sirius Red staining of myocardial cryosections from mice receiving HD AAV-Cre at 6 weeks of age. Note increased interstitial fibrosis in hearts of $NAE1^{F/F}$ mice. Scale bars, 100 µm. **D**, Echocardiographic measurements of mice at 4 weeks post AAV-Cre injection. EF, ejection fraction; FS, fractional shortening; LVID, left ventricle (LV) internal diameter; LVAW, LV anterior wall thickness; LVPW, LV posterior wall thickness; d, diastolic state; s, systolic state. **E**, Fast Green and Sirius Red staining of myocardial cryosections from mice receiving medium dose (MD) AAV-Cre at 4 weeks of age and quantification. Scale bars, 100 µm. **F**, TUNEL staining of indicated myocardium sections and the quantification of TUNEL+ cardiomyocytes. Scale bars, 100 µm. **G**, Confocal images of myocardium sections from hearts receiving HD AAV-Cre at 4 weeks and 6 weeks of age. Scale bars, 100 µm. Evans blue dye (EBD, 1%) was injected 24 hours before tissue collection. Myocardium sections from hearts collected at 24 hours after receiving a bolus dose of isoproterenol (ISO, 150 mg/Kg) were included as a positive control. Arrow heads indicate EBD positive signals. **H**, Western blot of cleaved CASPASE 3 (Cl. Casp3) and quantification. NS, not significant; *, $P<0.05$; **, $P<0.01$; ***, $P<0.001$; ****, $P<0.0001$. Error bars indicate SEM.

Supplemental Figure 2

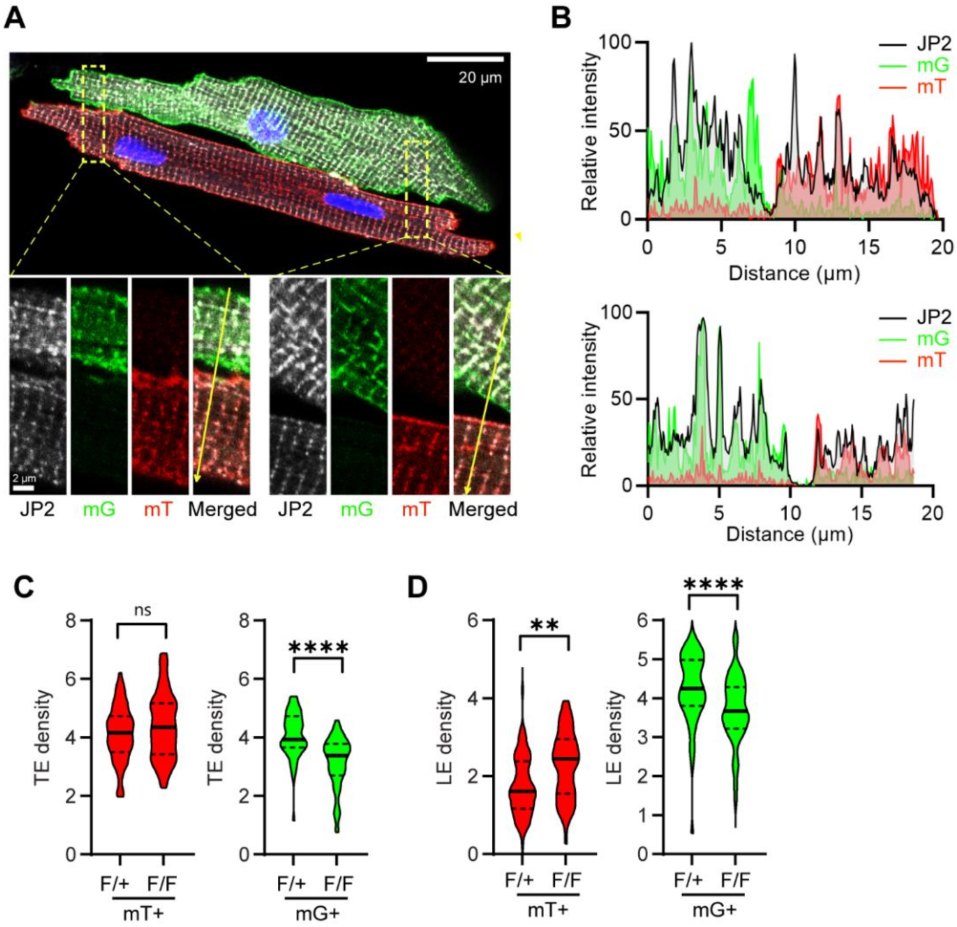

Supplemental Figure 2. Tracing T-tubule structure in cardiomyocytes using an mTmG reporter, Related to Figure 2. A, Immunostaining of adult CMs isolated from mTmG mice infected with AAV9-cTnT-Cre with JP2 antibody. Dashed boxes are enlarged in the bottom part. Note the colocalization of JP2 signals with mT and mG. Scale bars, 20 µm (top) and 2 µm (bottom). **B**, Quantification of relative fluorescent intensity across the yellow arrow in (**A**). **C-D**, quantification of transverse element (TE) density (**C**) and longitudinal element (LE) density (**D**) of T-tubules in CMs from hearts receiving LD AAV-Cre. *, *P*<0.05; **, P<0.01; ***, P<0.001; ****, P<0.0001; ns, not significant. Error bars indicate SEM.

Supplemental Figure 3

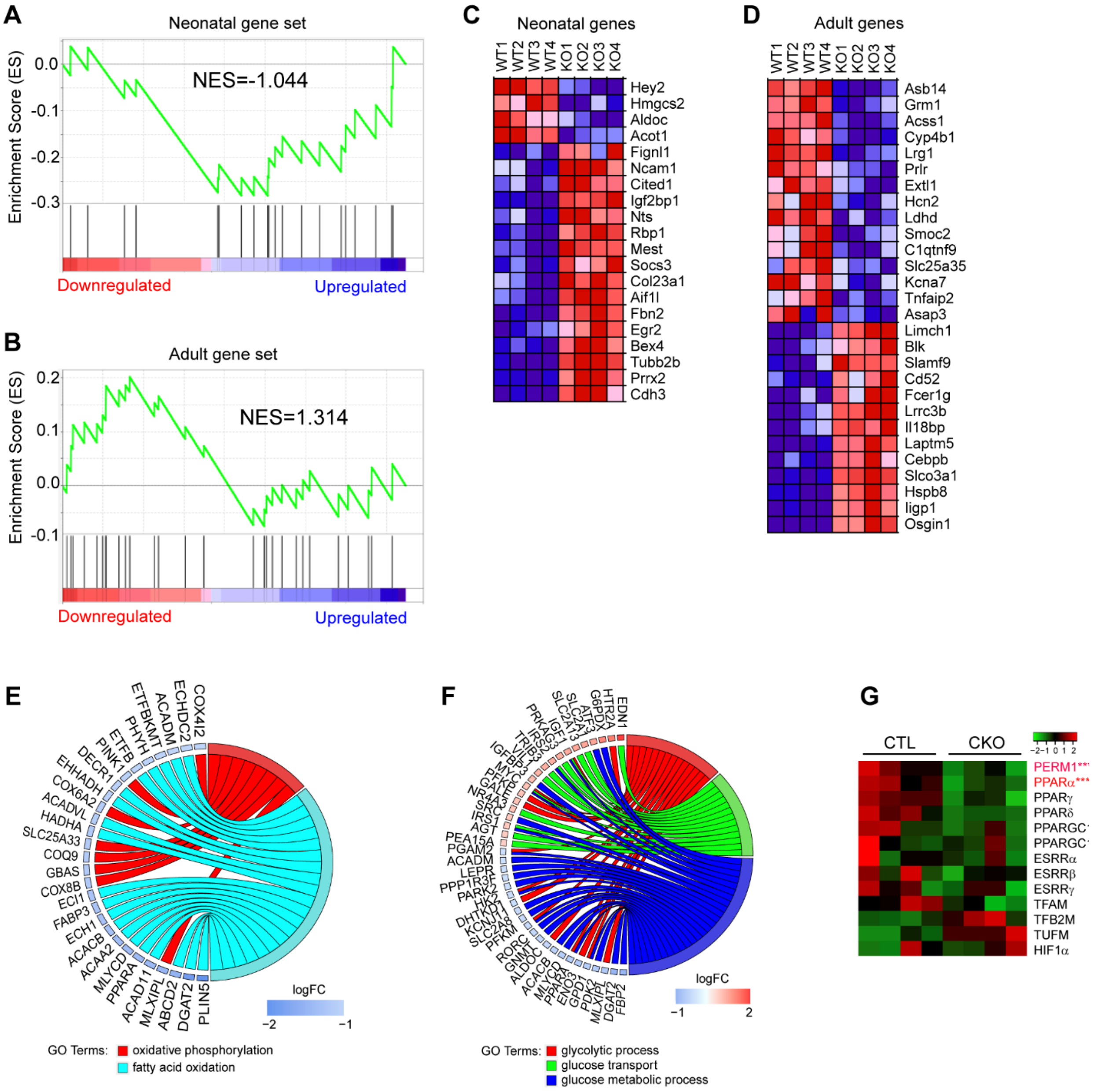

Supplemental Figure 3. Transcriptomic analysis reveals defective fetal-to-adult isoform switching and disrupted glycolytic and oxidative metabolism in NAE1-deficient hearts, Related to Figure 3. A-B, Gene set enrichment analysis of neonatal gene set (**A**) and adult gene set (**B**). NES, normalized enrichment score. **C-D**, Heatmap of specific genes that are enriched in neonatal gene set (**C**) and adult gene set (**D**). Blue, downregulated. Red, upregulated. **E-F**, Chord plot of genes within each GO terms as indicated by color code. **G**, Heatmap of indicated metabolic transcription factors. Green, downregulated. Red, upregulated. **, P<0.01; ***, P<0.001.

Supplemental Figure 4

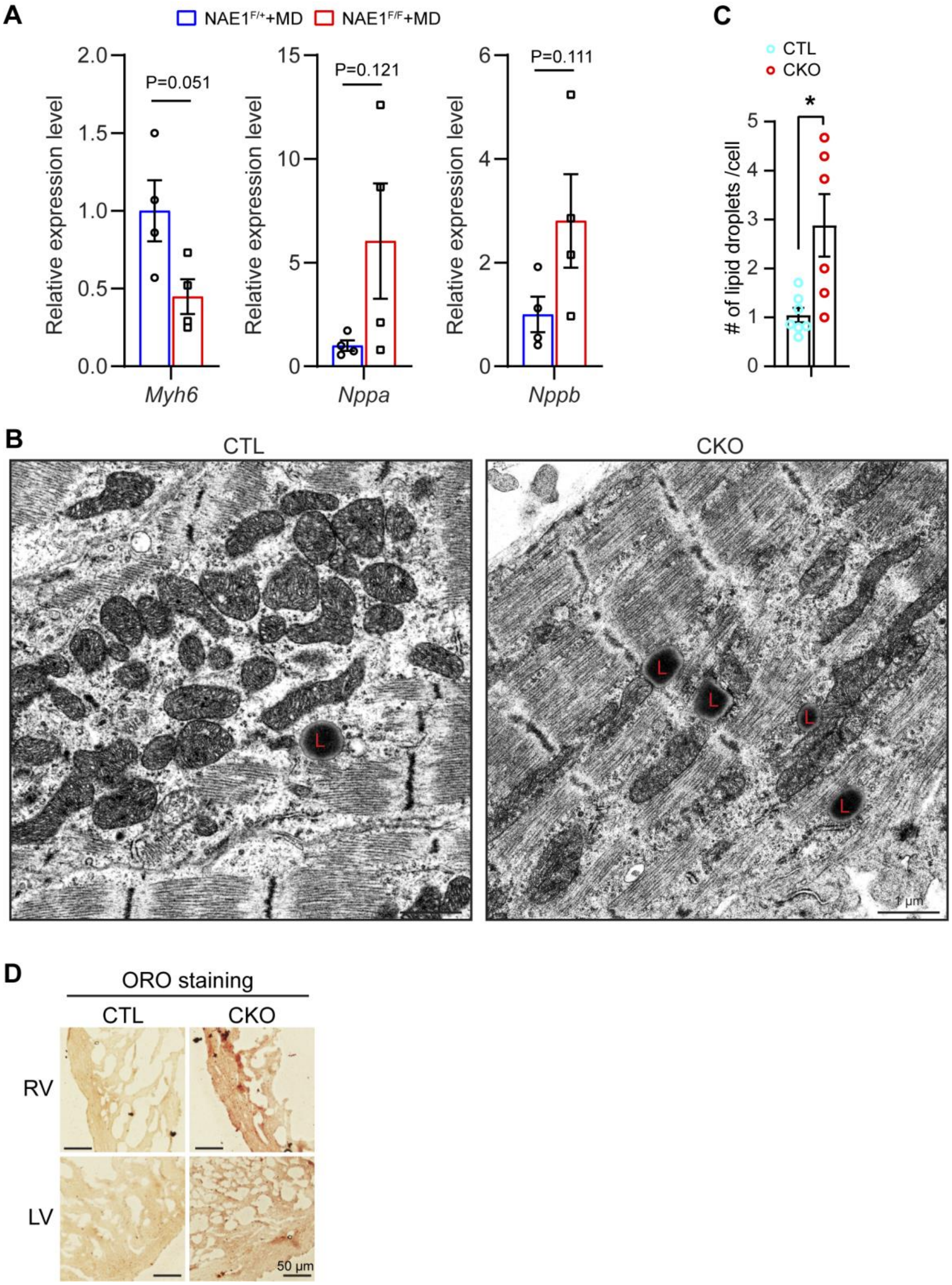

Supplemental Figure 4. The effects of NAE1 deficiency on the expression of cardiac stress markers and lipid levels, Related to Figure 4. A, QRT-PCR of indicated genes in hearts of mice infected with MD AAV-Cre. **B-C,** Representative electron microscopy images showing lipid (L) droplets in the hearts of neonatal control (CTL) and

NAE1^{CKO} (CKO) mice at P1 (Scale bars, 1 µm) and the quantifications ©. **D**, Oil-red O (ORO) staining of indicated heart tissues at P1. Scale bars, 50 µm. *, *P*<0.05; unless specified P-values. Error bars indicate SEM.

Supplemental Figure 5

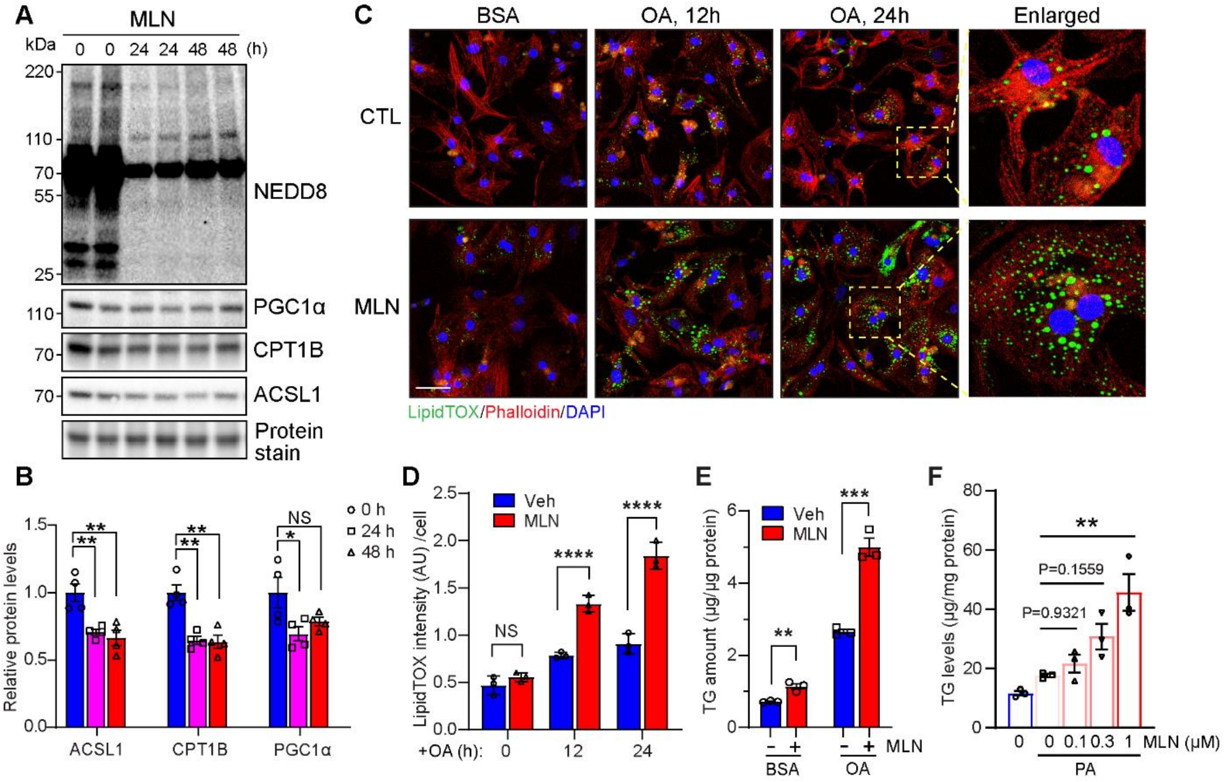

Supplemental Figure 5. Inhibition of neddylation by MLN4924 affects fatty acid metabolism in NRVCs, Related to Figure 5. A-B, NRVCs were treated with DMSO (Veh) or MLN4924 (MLN, 1 µM) for indicated times. Western blots (**A**) and quantification (**B**) of indicated proteins are shown. ACSL1, Acyl-CoA Synthetase Long Chain Family Member 1; CPT1B, Carnitine Palmitoyltransferase 1B; PGC1α, PPARG Coactivator 1 Alpha. **C-F**, NRVCs were treated with MLN (1 µM) for 48 hours. Oleic acid (OA, 100 mM) or Palmitate (PA, 100 µM) was added 24 hours unless specified before harvest. Representative confocal images of LipidTOX stained NRVCs (**C**, Scale bars, 50 µm.) and quantification (**D**) of LipidTOX fluorescent intensity per cell. n=3 replicates/group. **E-F**, Triglyceride (TG) levels in OA- or PA-treated NRVCs. NS, not significant; *, *P*<0.05; **, *P*<0.01; ***, *P*<0.001; ****, *P*<0.0001. Error bars indicate SEM.

Supplemental Figure 6

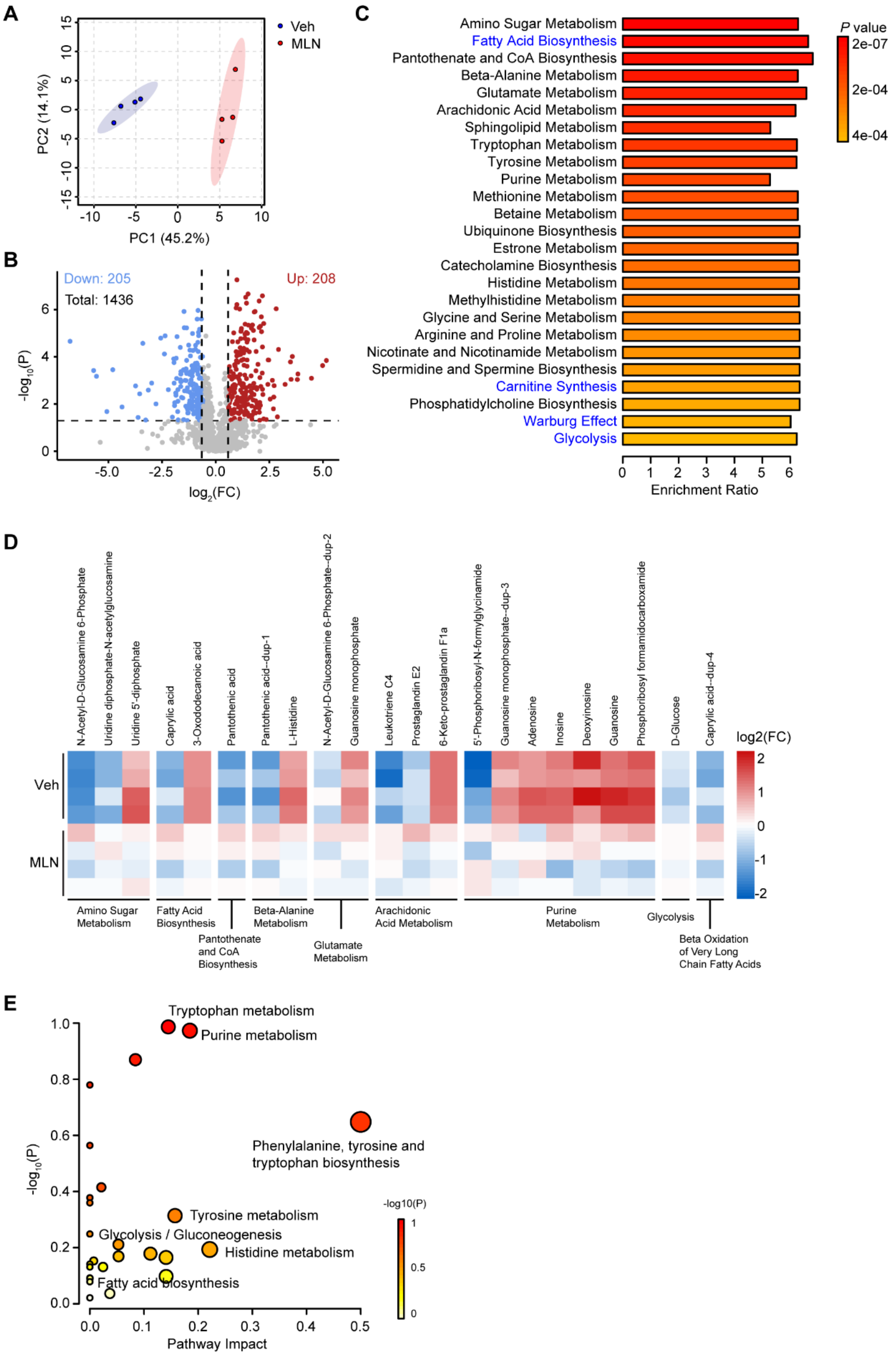

Supplemental Figure 6. Metabolomics analysis of MLN-treated NRVCs, Related to Figure 5. Untargeted metabolomics analysis of NRVCs treated with DMSO or MLN (1 µM) for 72 hrs (n= 4 biological replicates/group). **A**, Principal component analysis. **B**, Volcano plot showing significantly altered metabolites (FC>1.5 or <-1.5, *P* <0.05). **C**, Metabolite sets enrichment analysis. Bar length indicates enrichment ratio. Color degree indicates P value. **D**, Heatmap of significantly altered metabolites in sets in (**C**). **E**, KEGG pathway analysis of annotated metabolites. Color indicates -log10(P-value). Significantly enriched pathways are labeled in the plot.

Supplemental Figure 7

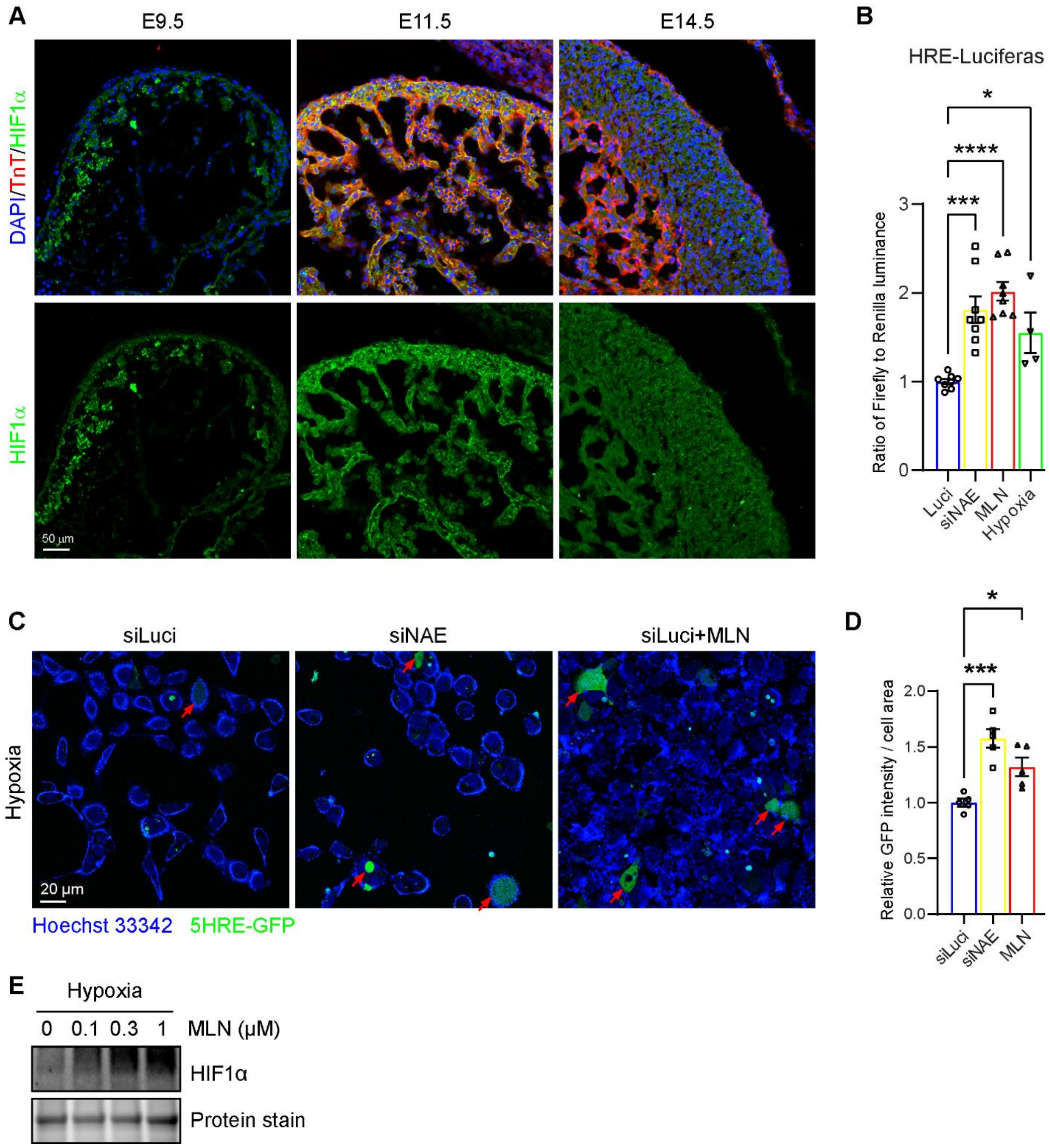

Supplemental Figure 7. Inhibition of neddylation leads to accumulation of HIF1α, Related to Figure 6 & 7. A, Immunostaining of HIF1α in myocardium cryosections from E9.5, E11.5, and E14.5 mouse embryos. Scale bars, 50 µm. **B**, H9C2 cells were transfected with plasmids expressing hypoxia response element (HRE)- dual-luciferases for 24 hours before treated with MLN (1 µM) or transfected with siRNA against NAE1 and UBA3 (siNAE) or against luciferase (siLuci) for additional 48 hours. The luminance of firefly luciferase was measured and normalized by that of Renilla to assess HIF1α activity. **C-D**, HEK293 cells were transfected with plasmids expressing HRE-GFP and treated as described in (**B**). Representative images (**C**, Scale bars, 20 µm) of GFP-expressing cells and quantification of GFP fluorescent intensity (**D**) are shown. **E**, Western blot showing HIF1α protein levels. NRVCs were treated with

the indicated doses of MLN treatment for 48 hours in a hypoxic (1% O_2) chamber. *, $P<0.05$; **, $P<0.01$; ***, $P<0.001$; ****, $P<0.0001$. Error bars indicate SEM.

SUPPLEMENTAL TABLES
Supplemental Table I. DEGs in NAE1CKO hearts_RNA-seq. Related to **Figure 3**.
Supplemental Table II. Metabolites in MLN-treated CMs. Related to **Figure 5**.
Supplemental Table III. Antibodies. Related to **STAR methods**.
Supplemental Table IV. Oligonucleotides. Related to **STAR methods**.
